# Clogging the Ubiquitin-Proteasome Machinery with Marine Natural Products: Last Decade Update

**DOI:** 10.3390/md16120467

**Published:** 2018-11-26

**Authors:** Gerardo Della Sala, Francesca Agriesti, Carmela Mazzoccoli, Tiziana Tataranni, Valeria Costantino, Claudia Piccoli

**Affiliations:** 1 Laboratory of Pre-Clinical and Translational Research, IRCCS-CROB, Referral Cancer Center of Basilicata, 85028 Rionero in Vulture, Italy; gerardo.dellasala@crob.it (G.D.S.); francesca.agriesti@crob.it (F.A.); carmela.mazzoccoli@crob.it (C.M.), tiziana.tataranni@crob.it (T.T.); 2 The NeaNat Group, Department of Pharmacy, University of Naples Federico II, via D. Montesano 49, 80131 Napoli, Italy; 3 Department of Clinical and Experimental Medicine, University of Foggia, via L. Pinto c/o OO.RR., 71100 Foggia, Italy

**Keywords:** natural products, ubiquitin, proteasome, marine, salinosporamide, cancer, high-throughput screening, secondary metabolites, lead compounds

## Abstract

The ubiquitin-proteasome pathway (UPP) is the central protein degradation system in eukaryotic cells, playing a key role in homeostasis maintenance, through proteolysis of regulatory and misfolded (potentially harmful) proteins. As cancer cells produce proteins inducing cell proliferation and inhibiting cell death pathways, UPP inhibition has been exploited as an anticancer strategy to shift the balance between protein synthesis and degradation towards cell death. Over the last few years, marine invertebrates and microorganisms have shown to be an unexhaustive factory of secondary metabolites targeting the UPP. These chemically intriguing compounds can inspire clinical development of novel antitumor drugs to cope with the incessant outbreak of side effects and resistance mechanisms induced by currently approved proteasome inhibitors (e.g., bortezomib). In this review, we report about (a) the role of the UPP in anticancer therapy, (b) chemical and biological properties of UPP inhibitors from marine sources discovered in the last decade, (c) high-throughput screening techniques for mining natural UPP inhibitors in organic extracts. Moreover, we will tell about the fascinating story of salinosporamide A, the first marine natural product to access clinical trials as a proteasome inhibitor for cancer treatment.

## 1. Introduction

Natural products (often referred to as secondary metabolites) have a long history as therapeutics for treatment of human diseases, including cancer. So far, most of the anticancer drugs currently used in therapy can be traced back to natural products (NPs), with the most striking examples being represented by terrestrial plant- or microbe-derived secondary metabolites such as vinblastine, taxol, and doxorubicin. More recently, chemical investigations of aquatic ecosystems (seas, oceans, lakes) have added novel chemical structures featuring antitumor properties. The great majority of these compounds were isolated from marine invertebrates, such as sponges, bryozoans, ascidians, and soft corals [1,2]. In particular, cytarabine, eribulin mesylate, trabectidin and brentuximab vedotin are either genuine marine NPs or marine natural product-derivatives, which are currently included in clinical settings for cancer treatment [3]. Moreover, 12 marine drugs are in different phases of clinical trials for clinical evaluation against different types of malignancies [4].

Drug development of natural lead compounds finds a limiting-step in the reduced access to enough quantities of pure compounds (the so-called supply problem), as secondary metabolites are usually produced in trace-amounts by marine organisms. The observation that many of these bioactive compounds are produced by associated microorganisms rather than the original source has moved attention towards exploration of the symbiotic microbiome, thereby leading to identification of microbial producers of bioactive molecules [5,6,7]. In parallel, researchers focused on the chemical profiling of free-living marine bacteria (mainly actinobacteria and cyanobacteria) and fungi, which are emerging as a sustainable ‘factory’ of natural products to fuel their clinical development [8,9].

Cancer cells are known to produce proteins, which are able to block cell-death pathways and/or induce cell proliferation and survival. With the discovery in the 1980s of the ubiquitin-proteasome pathway (UPP) as the central regulator of the intracellular-protein degradation machinery, preclinical, and clinical studies have explored proteasome inhibitors as anticancer drugs to shift the protein turnover towards cell-death [10]. Successful clinical trials led to Food and Drug Administration (FDA) approval of bortezomib, a first-generation proteasome inhibitor, and later to that of second-generation agents, including carfilzomib and ixazomib [10]. Notably, carfilzomib is an irreversible proteasome inhibitor inspired to epoxomicin, a naturally occurring peptidyl epoxyketone isolated from the Actinomycetes strain Q996-17 [11].

The UPP is an evolutionary well conserved pathway across eukaryotic species. It is well known that pathogenic bacteria have developed, among other strategies, the ability of producing secondary metabolites, to subvert and manipulate the host ubiquitin-proteasome system for their own benefits. Indeed, accumulating evidence suggests that bacteria produce proteasome inhibitors as virulence factors (e.g., syringolin A) [12]. In recent years, a plethora of secondary metabolites targeting the UPP pathway have been isolated from marine sources, particularly from marine sponges and sponge-associated microbial communities. Among marine invertebrates, soft corals, tunicates, and sponges are described as an unexhausted source of bioactive NPs. Marine sponges are microbial fermenters [13], as they are associated with symbiotic microorganisms, which for several sponge species contribute to 38–57% of the total biomass [14]. The soft coral reef is also a complex holobiont, resulting from coevolution of multiple macro- and microorganisms [2]. Therefore, these environments are a compelling arena where each microbial strain had to develop chemical signaling systems and secondary-metabolite biosynthetic machineries to (a) communicate with the host and other microbial species [15,16], (b) gain advantage over competitors [17], (c) defend itself or the host against predators [18], (d) retrieve nutrients from the environment [19].

In this scenario, natural UPP inhibitors from macro- and micro-organisms emerge as chemicals, which have been optimized during evolution to interact in a selective, potent manner with their target. As a consequence, NPs still represent an ideal starting point for drug development of novel UPP inhibitors.

So far, approved drugs targeting human proteasome feature serious side effects together with a restricted applicability and may even lose their efficacy due to the onset of drug-resistance mechanisms. Therefore, there is an urgent need to develop novel compounds with enhanced pharmaceutical properties to overcome these hurdles. In this review, we aim to (a) summarize the role of the UPP in anticancer therapy and (b) report about recent advances in discovery of natural UPP inhibitors from marine sources over the last decade, focusing on chemical structures and high-throughput screening techniques for mining natural UPP inhibitors. Moreover, we will tell about the fascinating story of the proteasome inhibitor salinosporamide A (marizomib, NPI-0052), providing an example of a successful drug-development pipeline, from natural product drug-discovery to clinical evaluation, through sustainable production by microbial fermentation.

## 2. The Ubiquitin-Proteasome Pathway as a Target for Cancer Therapy

The ubiquitin-proteasome pathway (UPP) is the central protein degradation system in eukaryotic cells, playing a key role in homeostasis maintenance through proteolysis of regulatory as well as misfolded (potentially harmful) proteins. For its function, the UPP intersects many cellular events, such as cell-cycle, apoptosis, cell survival, and DNA repair [20].

In cancer cells, there is an aberrant production of proteins inducing cell proliferation and/or inhibiting cell death pathways; these findings laid the fundaments to explore proteasome inhibition as an anticancer mechanism to shift the fine balance between protein synthesis and degradation towards cell death [10,21].

**Figure 1:**
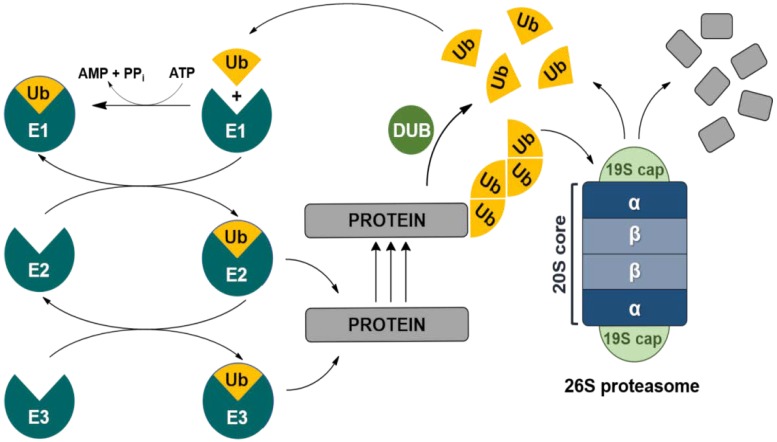
Protein degradation through the ubiquitin-proteasome pathway. The ubiquitin-activating enzyme E1 uses ATP to adenylate ubiquitin, yielding a ubiquitin-adenylate adduct and pyrophosphate. Ubiquitin is then transferred from E1 to the ubiquitin-conjugating enzyme E2. The ubiquitin ligase E3 transfers ubiquitin to a target substrate and a polyubiquitin chain is attached to tag the protein for degradation by the proteasome complex. Ubiquitin is released by deubiquitinating enzymes and the protein is degraded into oligopeptides. Ub, ubiquitin; ATP, adenosine triphosphate; ADP, adenosine diphosphate; AMP, adenosine monophosphate; PPi, inorganic pyrophosphate; DUB, deubiquitinase.

Intracellular protein degradation through the UPP involves three main steps (Figure 1): (a) polyubiquitination of the target protein for proteasome binding; (b) deubiquitylation of the bounded protein; (c) proteolytic digestion through the proteasome. Protein polyubiquitination is in charge of the ubiquitin-conjugating system, based upon interaction of ubiquitin-activating enzyme 1 (E1), ubiquitin-conjugating enzyme (E2), and ubiquitin-protein ligase (E3). Deubiquitinating enzymes (DUBs) detach polyubiquitin tag from the target protein, yielding monomeric ubiquitin (UB) to enclose a UB recycling circuit. Different cellular signals are generated by UB. Indeed, proteins are tagged for proteasomal degradation if they are conjugated to a polyubiquitin chain formed by linking UB molecules through lysine-48 residues. On the other hand, conjugation of a Lys 63-linked polyubiquitin chain or a single UB molecule to a substrate protein usually affects protein function and/or localization, regulating diverse cellular processes, such as receptor internalization, endocytosis, transcription, and DNA repair [22].

The proteasome, commonly referred to as the ‘26S proteasome’, is composed of two subunits: (a) the 19S particle, which mediate deubiquitylation in the constitutive proteasome and (b) the 20S core particle, responsible for protein degradation into oligopeptides. In the 20S core particle, three essential catalytic sites have been identified and termed after enzymes displaying similar functions: the chymotrypsin-like (β5 or ChT-L), trypsin-like (β2), and post-glutamyl peptide hydrolase-like (caspase-like or β1) activities.

With the FDA approvals (in 2003 and 2005) of the proteasome inhibitor bortezomib for treatment of relapsed/refractory multiple myeloma and mantle cell lymphoma [23], the UPP has been officially validated as a target of great interest for cancer therapy, providing a revolutionary way for treatment of malignancies.

**Figure 2:**
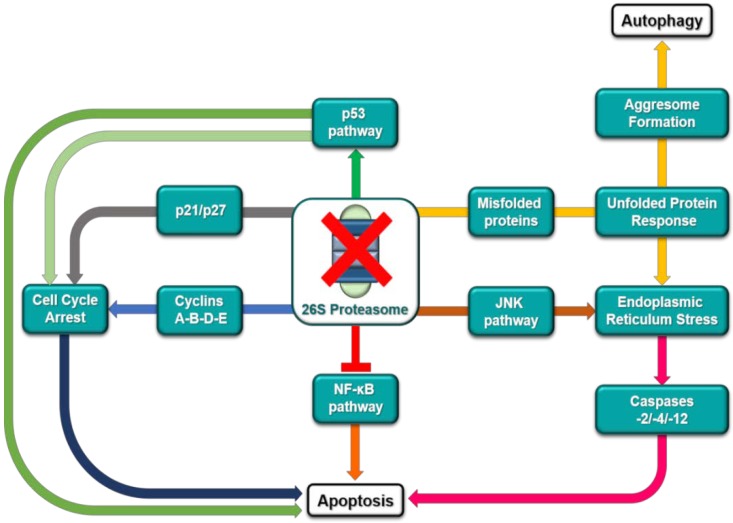
Key cellular targets affected by proteasome inhibition.

So far, it is known that proteasome inhibition induces apoptosis in cancer cells via different concurrent mechanisms (Figure 2). Impairing proteasome activity results in the inhibition of IkB degradation and, consequently, leads to inactivation of the nuclear factor NF-κB, which is a tumor-promoting and antiapoptotic transcription factor [24]. Beyond its effect on NF-κB, proteasome inhibition may (a) prevent degradation of proapoptotic proteins, such as the tumor suppressor protein p53 and the cell-cycle regulator p27, (b) reduce levels of antiapoptotic proteins (e.g., Bcl-XL, Bcl-2, and STAT-3), and (c) enhance the c-Jun N-terminal kinase (JNK) pathway, thus promoting a proapoptotic state [25,26,27]. Furthermore, decreased levels of proteasome activity generate an imbalance between proteasome load and its digestion capacity, resulting in accumulation of polyubiquitinated proteins and reduction of UB recycle [28]. This condition, known as proteasomal stress, has been regarded as a proapoptotic signal within the cell. In parallel, accumulation of misfolded proteins in the endoplasmic reticulum and cytoplasm triggers the unfolded protein response (UPR), a stress signaling pathway which tries to compensate proteasome deficiency, through reduction of protein synthesis and production of folding chaperones and proteasome components (Figure 2). However, in protracted stress condition, as during treatment with proteasome inhibitors, UPR cannot guarantee cell survival and leads to cell cycle arrest and apoptosis [10,29]. Besides the previously described apoptotic effects, downregulation of proteasome and unprocessed deleterious proteins may also promote aggresome formation, excessive autophagy and, therefore, cell death (Figure 2) [30].

In recent years, drug discovery programs in academia and pharmaceutical companies have been addressed towards inhibitors of the UPP pathway for cancer treatment. Beyond the proteasome itself, enzymes such as E1, E2, E3, and DUBs have emerged as druggable targets for designing new and more selective generations of antitumor compounds.

Blocking the ubiquitination pathway at the level of the ubiquitin-activating enzyme E1 (UBE1) may potentially impair the great majority of the UB-dependent cellular processes. Knockdown of UBE1 as well as its chemical inhibition has been associated to decreased levels of ubiquitylated proteins within cells and to cell death in leukemia rather than normal cells. In addition, inhibition of this E1 enzyme delayed tumor growth in a mouse model of leukemia [31]. Mechanistically, UBE1 inhibition induced cell death mainly by prompting endoplasmic reticulum stress and the UPR response (Figure 2). However, like proteasome inhibition, increased levels of p53 and inhibition of tumor necrosis factor-α-induced activation of NF-κB have been observed after UBE1 inhibition. In addition to UBE1, the NEDD8 activating enzyme (NAE) has attracted attention due to its role in regulation of the SCF-E3 ligase activity through neddylation. Blocking NAE-mediated neddylation results in deactivation of SCF, leading to cell cycle arrest and apoptosis, through the accumulation of p27, NRF2, CDC25A, HIF1α, and IκB [32].

To date, more than 40 variants of the E2 enzyme have been discovered to be encoded by human genome. These enzymes are able to regulate the switch from ubiquitin chain initiation to elongation, the processivity of chain formation and the topology of assembled chains, thereby determining fate and/or function for the modified proteins. Among members of the E2 enzyme-superfamily, the ubiquitin-conjugating enzyme Ubc13 forms a complex with Uev1A or MMS2 to ubiquitinate p53, preventing p53 tetramerization and, consequently, decreasing its transcriptional activity. Therefore, hampering the formation of Ubc13/Uev1A complexes leads to p53 activation, arresting cell proliferation and survival (Figure 2). Indeed, knockdown of Ubc13 expression in OCI-Ly10 cells is accompanied by increased expression of p21 and GADD45, which are known to be p53 targets [33]. More interestingly, it has been reported that overexpression of the ubiquitin-conjugating enzyme E2C (UBE2C) is a typical feature of a number of cancer cell lines and primary tumors, including lung, gastric, bladder, and uterine cancers. RNA-silencing of UBE2C, which is involved in ubiquitination of cell-cycle regulators, caused antiproliferative effects and cell-cycle deregulation in esophageal adenocarcinoma cells [34].

So far, approximately 600 variants of the E3 enzyme have been identified, each of them being responsible for binding and ubiquitylating a defined set of substrate proteins. Therefore, it can be conceived that inhibition of a specific E3 isoform affects a well-defined cellular pathway. The E3 MDM2 (or HDM2) is the main regulator of the p53 tumor suppressor protein degradation process; as a result, blocking MDM2 activity leads to p53 stabilization and increased anticancer activity of p53 downstream effectors (Figure 2) [35]. In a similar way, inhibition of the E3 ITCH guarantees stabilization of p63 and p73, which are known to share several target genes with p53. Furthermore, it has been reported that several malignancies feature downregulation of the cell-cycle inhibitor p27, which can be restored through inhibition of its degradation pathway governed by the oncogenic E3 ligase Skp2 (Figure 2) [35].

DUBs are enzymes involved in removal of the UB-degradation signal from proteins, regeneration of cellular UB pools, and editing of polyubiquitin tag on specific substrates to address their fate. Among them, UCHs and USPs are the best characterized, with USPs representing more than half of the known human DUBs. The main function of the variant USP7 is the indirect regulation of p53 homeostasis [36]. USP7 inhibition blocks deubiquitylation of MDM2, which is exposed to intensive proteasome degradation. Decreased levels of MDM2 stabilize p53, prolonging its beneficial anticancer effects (Figure 2) [36].

To date, bortezomib, carfilzomib, and ixazomib have received FDA regulatory approval and have been introduced as proteasome inhibitors in clinical settings. In addition, thalidomide and the more novel analogues lenalidomide or pomalidomide have been approved for treatment of multiple myeloma, relying on their selective action against the E3 ubiquitin ligase cereblon [37].

In this contest, natural product drug-discovery has provided a precious contribution to the search for anticancer lead compounds targeting the UPP pathway. Particularly, exploration of marine environments is adding novel chemical entities for drug designing, which have been isolated from either invertebrate (sponges, tunicates, algae, soft corals) or microbial (actinobacteria, cyanobacteria, fungi) sources. At present, the most interesting story is that of the marine natural product salinosporamide A (**1**), which has entered phase III clinical trials, due to its strong biological activity as irreversible proteasome inhibitor.

## 3. Salinosporamide A, a Milestone in Natural Product Drug Discovery

Salinosporamide A (**1**) as well as several structural analogues (salinosporamides B-K, **2**–**11**) have been isolated from marine obligate actinomycetes of the genus *Salinospora*. The salinosporamide family displays a typical γ-lactam-β-lactone structural moiety; in **1**, there are a chloroethyl side chain and a cyclohexenyl unit at the α positions of the lactam and lactone rings, respectively.

**Figure 3:**
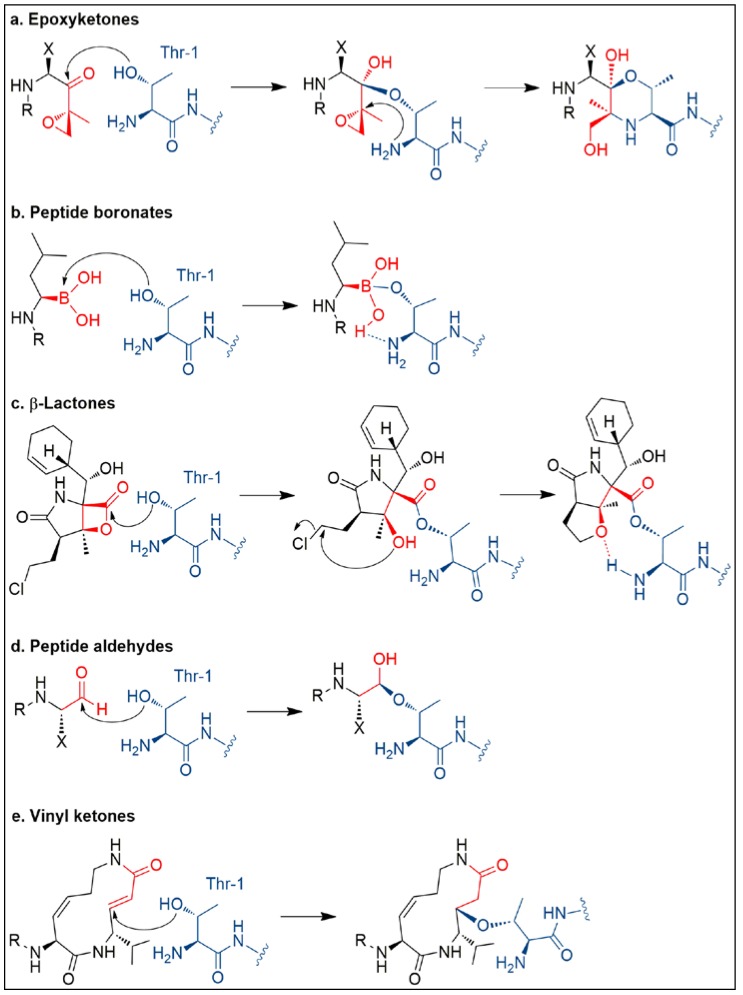
Mechanism of action of proteasome inhibitors. (**a**) Epoxyketones react with the N-terminal threonine (Thr-1) of the catalytically active β-subunits in a two-step mechanism. Initially a 1,2-addition of the γ-OH group of Thr-1 (Thr1Oγ) to the carbonyl group of the epoxyketone leads to the formation of a hemiketal derivative; then, the α-amino-group of Thr-1 makes a nucleophilic attack at C-2 of the epoxide moiety to give a stable morpholine adduct. (**b**) In peptide boronates, the boron atom covalently interacts with the nucleophilic oxygen lone pair of Thr1Oγ, yielding a tetrahedral boronate adduct. This adduct is in turn stabilized by an acidic boronate OH group, which hydrogen-bridges Thr-1 amine atom. (**c**) The side-chain hydroxy group of Thr-1 cleaves the lactone ring of β-lactone proteasome inhibitors, yielding the proteasome-inhibitor complex; then, in the case of salinosporamides, the newly formed tertiary alcohol displaces the chlorine atom, leading to the formation of a tetrahydrofuran ring, which in turn coordinates the α-amino group of Thr-1. (**d**) The carbonyl moiety of peptide aldehydes is the electrophilic site which undergoes nucleophilic attack by Thr-1 to form an hemiacetal adduct; (**e**) Vinyl ketones harbor a Michael system which undergoes 1,4-nucleophilic addition with the Thr1Oγ, forming a one-step irreversible covalent adduct.

**Figure 4:**
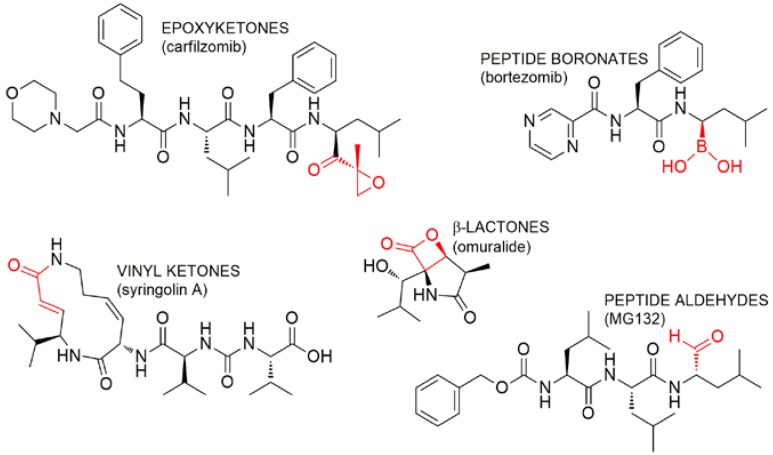
The five classes of proteasome inhibitors. Proteasome inhibitors possess an electrophilic group for covalent binding to the proteasome. The reactive pharmacophore of each inhibitor is highlighted in red.

The unique interaction of **1** with all three β catalytic sites of the 20S proteasome was elucidated through X-ray crystallographic analysis [38]. The molecular mode of action (Figure 3c) can be summarized in three sequential steps: (1) initially, as it has been reported for other γ-lactam-β-lactone proteasome inhibitors (e.g., omuralide, Figure 4), the side-chain hydroxy group of the N-terminal threonine residue (Thr1) of the proteasome cleaves the lactone ring of **1**, yielding the proteasome-inhibitor complex; (2) then, the newly formed tertiary alcohol of **1** displaces the chlorine atom, leading to the formation of a tetrahydrofuran (THF) ring, by an intramolecular nucleophilic substitution mechanism; (3) finally, the THF ring prevents hydrolysis of the ester bond between proteasome and **1** (a) by displacing water molecules and coordinating the α-amino group of Thr1 and (b) by blocking reformation of the β-lactone ring. This unique interaction mode makes **1** an irreversible inhibitor, tightly bounded to the 20S proteasome. In addition, the cyclohexenyl unit is essential both to shield the ester bond of the proteasome-inhibitor complex and to establish favorable hydrophobic interactions in the binding pocket. These interactions are not possible for other known γ-lactam-β-lactone proteasome inhibitors and therefore could be the reason why **1** is able to recognize all three β catalytic sites of the 20S proteasome [38].

Salinosporamide A is able to inhibit the proteasome in the low nanomolar range and is the most active among the members of the salinosporamide family. Natural variants of **1** mainly differ for modifications at the C-2 side chain, with few exceptions being represented by the decarboxylated pyrrole analogue salinosporamide C (**3**), the C-3 ethyl analogue salinosporamide I (**9**), and the non-hydroxylated derivative salinosporamide J (**10**).

Evaluation of biological activity of natural and non-natural salinosporamide variants were useful to fully understand their mechanism of action and reveal structure-activity relationship (SAR) features. SAR studies pointed out that the presence of a good leaving group at the C-2 side chain is essential to exert a strong activity against all the β subunits as it assists the THF ring closure, which stabilizes the proteasome-inhibitor complex (Figure 3c) [39,40]. Good leaving groups such as halides (Cl^−^, Br^−^, and I^−^) or sulfonate esters (tosylate, dansylate, mesylate) are well tolerated and can be easily accommodated in the large S2 binding pocket of the proteasome (Figure S1). Accordingly, non-leaving groups decrease dramatically drug efficacy. These observations were confirmed by the interesting case of fluorosalinosporamide (**12**), which has been prepared by genetic manipulation of the original organism in B. S. Moore’s laboratory to introduce fluoride instead of chloride as leaving group [41]. The poor leaving group ability of fluoride and the subsequent slow displacement of the F atom during proteasome-inhibitor interaction is in accordance with the behavior of fluorosalinosporamide as a partially reversible proteasome inhibitor, thus confirming the mechanism of action discussed above. As a result, modification of leaving group ability at C-2 allows fine-tuning of proteasome inhibition by salinosporamides. In addition, it is worthy noting that C-2 stereochemistry of the natural compound must be retained to keep a strong bioactivity profile as the natural C-2 epimer of **1**, salinosporamide F (**6**), displayed an about 100-fold weaker activity [39,42].

As regards substitutions of the cyclohexenyl unit at C-5, it has been demonstrated that any C-5 analogue exerted attenuated activity, with exception of the synthetic cyclopentenyl derivative, which exhibited a bioactivity comparable to **1** (Figure S1) [43]. The minor metabolite antiprotealide (**13**) from *Salinospora tropica* has an isopropyl chain at C-5 and displayed a 50-fold lower inhibitory effect than **1**. Overall, linear substituents at C-5 reduced bioactivity, probably due to the destabilizing ‘waving’ effect of a more flexible chain in the S1 binding pocket of the proteasome [43].

Further alterations of the natural scaffold of **1**, such as the substitution of the methyl group at C-3 (e.g., salisporamide I (**9**)) or removal of the hydroxy group at C-5 (e.g., salisporamide J (**10**)) are always associated with a weakened biological effect [40,42]. Taken together, SAR studies showed that salinosporamide A is the strongest proteasome inhibitor among natural and synthetic members of this chemical class, thus highlighting a molecule perfectly designed by nature, which placed every single atom at the right position to optimize interactions with the target [44].

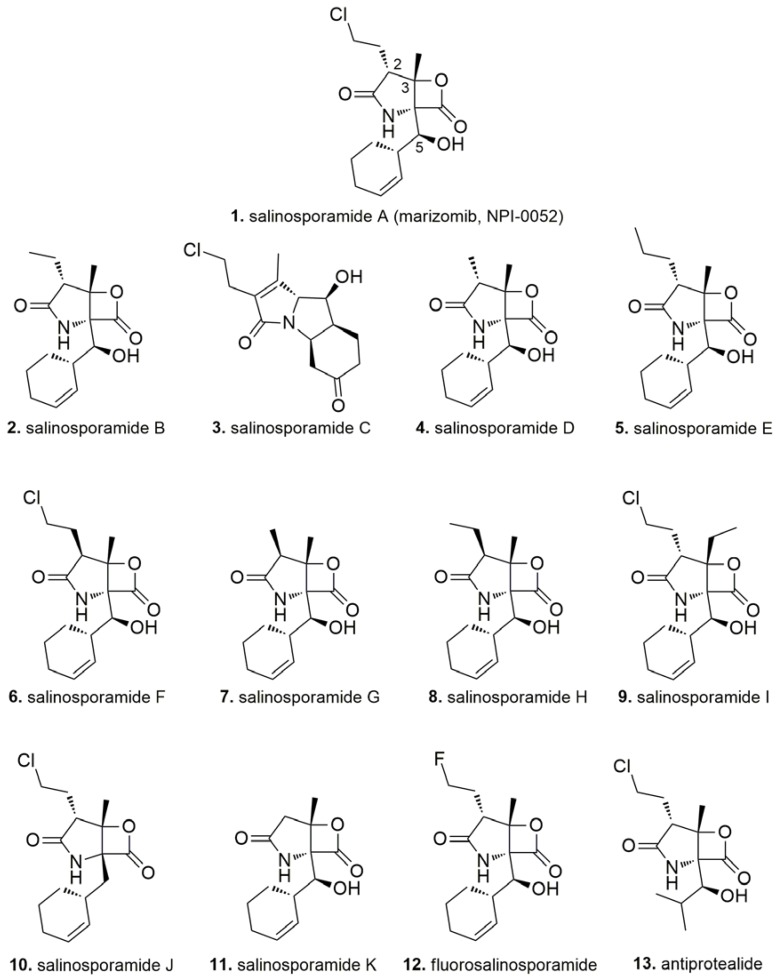



Besides its established activity as a pan-proteasome inhibitor, **1** has been demonstrated to (a) enhance apoptosis, suppress osteoclastogenesis, and inhibit invasion through suppression of the NF-κB pathway [45]; (b) interfere with NF-κB-Snail-RKIP causing downstream inhibition of antiapoptotic gene products and epithelial to mesenchymal transition [46,47]; (c) induce caspase-8 and ROS-dependent apoptosis in leukemia cells [48].

Preclinical studies showed the increased efficacy (=increased potency and longer duration of biological effects) of **1** compared with bortezomib [45]; this observation together with an attenuated toxicity at therapeutic doses allowed **1** to enter the clinical development process in 2006 with a phase I trial (NCT00396864) in patients with advanced solid tumor malignancies or refractory lymphoma whose disease had progressed after treatment with standard therapies. Since then, four additional phase I/II trials were completed to examine safety, pharmacokinetics, and pharmacodynamics of **1** in patients with (a) relapsed or relapsed/refractory multiple myeloma (NCT00461045); (b) solid tumors, lymphoma, chronic lymphocytic leukaemia (CLL), Waldenstrom’s macroglobulinemia, and relapsed and/or refractory (RR) multiple myeloma (NCT00629473); (c) melanoma, pancreatic carcinoma or non-small cell lung cancer, combining **1** and the HDAC inhibitor vorinostat (NCT00667082); (d) relapsed and refractory multiple myeloma, combining **1**, pomalidomide, and low-dose dexamethasone (NCT02103335) [49,50,51]. Furthermore, salinosporamide A was shown to exert a selective cytotoxic action against malignant glioma stem cells and glioma cell lines with minimal effect on neural stem cells [52]. Therefore, three clinical studies were recently undertaken and are still in progress to evaluate (a) a new combination of drugs, **1** and bevacizumab (BEV; Avastin^®^, Genentech Inc., South San Francisco, CA, USA), for the treatment of WHO Grade IV malignant glioma (NCT02330562); (b) the addition of **1** to standard-of-care treatments of radiotherapy (RT), temozolomide (TMZ), and Optune in patients with newly diagnosed WHO Grade IV malignant glioma (NCT02903069); (c) efficacy of **1** in newly diagnosed glioblastoma patients (phase III trial, NCT03345095).

Supply of natural product leads usually hampers their full utilization for clinical development, as marine natural products are often isolated in trace quantities from original sources. In order to overcome these hurdles and ensure development of **1**, efforts were addressed towards enhanced production of **1** through optimization of an industrial-scale saline fermentation process, meeting Current Good Manufacturing Practice (CGMP) guidelines. Indeed, the compound is currently obtained by cultivation of the natural producer *Salinospora tropica*, despite more than 10 synthetic strategies have been developed to produce **1** along years [53]. This is the first example of a GMP saline fermentation process employed to produce a natural-product drug from a marine microbe, highlighting a feasible path for drug development from marine natural products.

## 4. Inhibitors of the Ubiquitin-Proteasome Pathway in the Last Decade (2007–2018)

Marine organisms and microorganisms are able to assemble complex biosynthetic machinery to produce pharmacologically-active compounds having unusual architectures with no counterparts in the terrestrial environment. Indeed, natural products from marine sources still represent a source of inspiration for anticancer drug discovery, where fuelling new ideas is constantly needed.

With the advent of MS and NMR instrumentations for nanoscale structure elucidation [54,55] and advanced cell- and protein-bioassays, a plethora of secondary metabolites with fascinating chemical structures and potent cytotoxic properties have emerged from aquatic environments.

Most UPP inhibitors known to date interact specifically with the proteasome. Proteasome inhibitors usually feature an electrophilic group to allow covalent binding to the proteasome by nucleophilic attack of the Thr1 hydroxy groups of the proteolytic β subunits (Figure 3 and Figure 4). Based upon the different chemical nature of the electrophilic site, proteasome inhibitors can be classified in five groups (Figure 4), including peptide boronates (e.g., bortezomib), peptide aldehydes (e.g., MG132), macrocyclic vinyketones (e.g., syringolin), β-lactones (e.g., omuralide, salinosporamides), and epoxyketones (e.g., carfilzomib).

Herein, we aim to review the chemical diversity of natural products from marine sources identified as UPP inhibitors in the last decade, reporting about not only proteasome, but also E1, E2, E3, and DUBs inhibitors. We grouped the reviewed compounds according to the canonical structural classification of natural products.

### 4.1. Terpenes

A bioassay-guided investigation of the crude extract of the Philippines sponge *Acanthodendrilla* sp. led to the isolation of the proteasome inhibitor acanthosulfate (**14**). Acanthosulfate is a merosesterterpenoid possessing a scalarane-type carbon skeleton, with an unusual *trans-antitrans-syn-anti* configuration. Although showing an IC_50_ value of 4.5 µM in a proteasome inhibition assay, this compound was found to be unselective and ineffective in the BMS Oncology Diverse Cell Panel, and therefore any further development was stopped [56].

An in-depth analysis of the methanolic extract of *Rhabdastrella globostellata* (South China Sea) revealed the presence of nine new isomalabaricane-derived natural products, globostelletins A-I (**15–23**), together with some known metabolites, such as jaspolides F (**24**), rhabdastrellic acid A (**25**), and stellettin E (**26**). Isomalabaricanes are a class of sponge-specific *trans-syn-trans* 6,6,5-tricyclic triterpenoids. In vitro evaluation of cytotoxicity of **15**–**26** on a panel of five different cell lines highlighted selective inhibitory activity against human ovarian carcinoma A2780 cell line, with the most potent compounds being represented by **25** and **26**. Particularly, drug efficacy (a) increased with the length of the side-chain and the number of conjugated double bounds and (b) was higher for 13-*Z* than the 13-*E* isomers [57]. In this work, Li et al. focused their attention on rhabdastrellic acid A, observing a similar antiproliferative effect against both A2780 cells and human promyelocytic leukemia cell line HL-60. Rhabdastrellic acid A was shown to induce apoptosis in HL-60 cells and cell-cycle arrest in the G2/M phase through upregulation of p21. This effect is likely related to the proteasome inhibitory effect of rhabdastrellic acid A, thus blocking the pathway for p21 degradation [57]. Notably, in a proteasome activity assay, β5 and β2 catalytic sites of the proteasome were equally inhibited by **25**, with a rate >80% at a concentration of 30 μM. The UPP pathway could be also damaged by ROS accumulation, deriving from rhabdastrellic acid A-induction of caspase-3 in HL-60 cells. Surprisingly, in another study based upon different cell models (hepatic Hep3B and lung A549 cancer cells), **25** did not cause apoptosis but it was found to induce autophagy-associated cell death through blocking of Akt pathway [58].

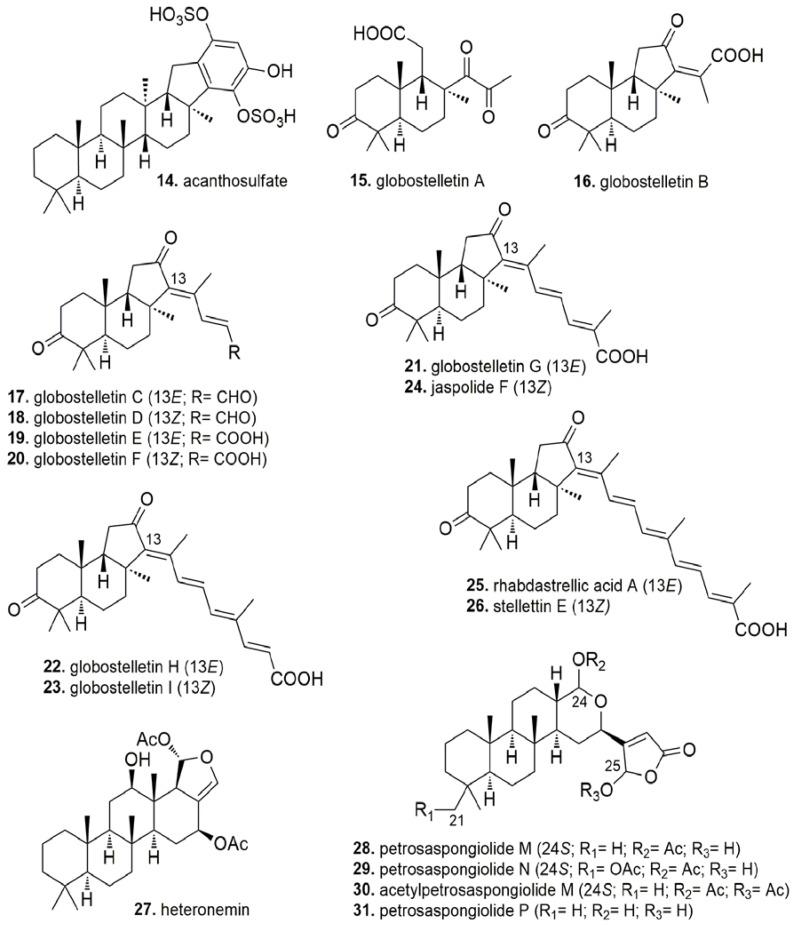



The scalarane-type sesterterpene heteronemin (**27**) was isolated for the first time in 1976 from the sponge *Heteronema erecta* (Vanuatu, South Pacific), but its mechanism of action was investigated only in the last decade [59]. In chronic myeloid leukemia K562 cells, heteronemin was shown to affect several pathways involved in cell proliferation and survival, including apoptosis, cell-cycle arrest, autophagy, the MAPK signaling pathway, and the inhibition of the NF-κB-dependent system. With regards to cell death, **27** was able to induce apoptosis triggering caspases-8- and -9-dependent pathways. The heteronemin-inhibitory effect of NF-κB may play a key role in inducing a proapoptotic state within the cells. At a molecular level, it was demonstrated that **27** blocked TNFα-activation of NF-κB signaling, preventing IkBα degradation and phosphorylation through inhibition of the 26S proteasome, without affecting IKK activity. All the three proteolytic sites of the proteasome were inhibited by **27**, with IC_50_ values of 0.4 μM for the chymotrypsin- and trypsin-like catalytic sites, and >2.8 μM for the caspase-like site.

Petrosaspongiolide M (**28**) is a marine sesterterpene (*Petrosaspongia nigra*, New Caledonia) with a γ-hydroxybutenolide ring, showing a significant ability to inhibit the proteasome activity both in enzymatic and cell-based assays. Particularly, in cell human-macrophage-derived THP-cell line, **28** inhibited the caspase- and the chymotrypsin-like activity with IC_50_ values of 0.85 μM and 0.64 μM, respectively, while the IC_50_ of the trypsin-like activity was >1 μM [60]. Margarucci et al. identified the molecular target of **28**, applying a chemical proteomic strategy based upon combination of affinity chromatography and LC/MSMS spectrometry. Briefly, macrophage cell lysates were loaded on a solid support functionalized with **28**, and, after extensive washing, anchored proteins were released and analyzed by mass spectrometry. In addition, as a consequence of proteasome inhibition, a significant accumulation of polyubiquitinated proteins could be detected by western blotting after treatment of THP-1 cells with **28**. In an attempt to outline SAR profile of **28**, Margarucci et al. evaluated modulation of proteasome machinery by several natural and synthetic analogues of **28** in vitro and cell-based bioassays [61]. Overall, minor modifications in the natural analogues—such as acetylation at C21 and C24 in petrosaspongiolide N (**29**), acetylation at C25 in acetyl-petrosaspongiolide M (**30**), and deacetylation at C24 in petrosaspongiolide P (**31**)—resulted in weakened bioactivity. Synthetic analogues of **28** were prepared preserving the γ-hydroxybutenolide ring and replacing the terpene moiety with a number of aromatic systems. All modifications were ineffective, except for the benzothiophene ring, which was shown to be a valuable alternative to the terpene moiety only in enzymatic assays (Figure S2). Indeed, **28** still revealed to be the most bioactive compound in cell-based assays, suggesting the terpene scaffold to play an essential role in proteasome recognition and/or molecular adsorption/transport through cell membranes. Notably, the masked aldehyde function at C25 of **28** is critical for bioactivity and represents a putative reactive site for covalent binding to the proteasome, as acetylation of C25 lowered dramatically the inhibitory effects in natural and synthetic analogues. Additionally, a close correlation between accumulation of polyubiquitinated proteins and the number of apoptotic cells was clearly observed after treatment of human monocytic U937 cells with **28**. Afterwards, in-depth investigation of the molecular mechanism of **28** was undertaken, unveiling its ability to impair complementary pathways involved in protein degradation, namely the UPP and the autophagy systems [62]. Mass-spectrometry data analysis integrated with molecular docking studies, highlighted that **28** exerted its proteasome inhibitory activity by binding the active sites in the inner core of the immunoproteasome and/or covalently binding a Lys residue of the 11S proteasome activator. The immunoproteasome is a variant of the 26S constitutive proteasome; it is constitutively expressed in hematopoietic cells and induced in non-immune cells, being involved mainly in immune response regulation, activation of the NF-κB pathway, and management of oxidative stress. Upon U937 cells treatment with 1 μM of **28**, all components of the immunoproteasome as well as the 26S ChT-L activity were significantly depressed. Moreover, **28** induced depletion of Beclin-1 intracellular levels and accumulation of LC3II and p62, providing strong evidence for impairment of the autophagy-mediated proteolysis.

The EtOAc extract of the marine sponge *Petrosia corticata* (North Sulawesi, Indonesia) revealed a relevant proteasome inhibitory activity due to the presence of bioactive meroditerpenoids, known as strongylophorines (**32**–**39**) [63]. Chemical purification of this fraction led to the isolation of two novel strongylophorines (**32**–**33**) together with six known analogues. In vitro evaluation of inhibition of the proteasome ChT-L catalytic site allowed to identify a mixture of strongylophorines-13 and -14 (**38**) as the most potent bioactive compound (IC_50_ = 2.1 μM). In general, the combination of the hydroquinone moiety with the hemiacetal functionality in **38** depicted the best pharmacophore within these natural compounds. Replacement of the hemiacetal function with an acetal (**32** and **33**) or a lactone group (**34** and **37**) resulted in weakened inhibitory effects. At the same time, the hydroquinone-containing metabolites were more effective than the corresponding dehydrated analogues. Abolishment of the hemiacetal group with the introduction of a carboxylic acid (**35**), aldehyde (**36**), and methyl group (**39**) at C-4 was shown to decrease inhibitory activity in this order.

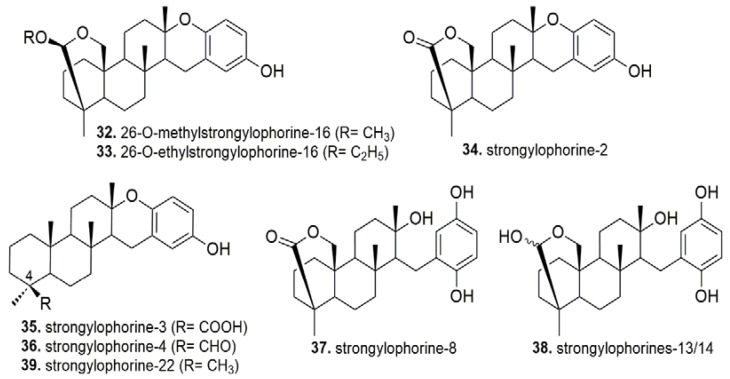



Sulawesin A (**40**) and B (**41**), along with the two known ircinin-1 (**42**) and -2 (**43**), were isolated from a *Psammocinia* sp. marine sponge (Indonesia) and identified as inhibitors of the ubiquitin-specific protease 7 (USP7). Structurally, these compounds are furanosesterterpenes with a tetronic-acid moiety. **40**–**43** were found to be active in vitro in the low micromolar range, with IC_50_ values of 2.8, 4.6, 2.7, and 3.5 μM, respectively [64].

Niphateolide A (**44**) was isolated from the sponge *Niphates olemda* (Indonesia) as a stereoisomeric mixture at C-17. The structure of **44** has a diterpene scaffold with a γ-hidroxybutenolide moiety [65]. This compound was found to act as an inhibitor of the HDM2-p53 interaction by ELISA (IC_50_ = 16 μM).

A family of sequiterpene cyclohexenones, namely epoxyphomalins A–E (**45**–**49**), was isolated from the marine-derived fungus *Paraconiothyrium cf sporulosum*, obtained from the Caribbean sponge *Ectyplasia perox* [66,67]. Dissecting epoxyphomalin chemical structure, it can be deduced these compounds are made of a polyketide-derived epoxydon moiety and an isoprenoid-derived decalin ring. The cytotoxicity of compounds **45**–**49** was investigated using a monolayer cell survival and proliferation assay in a panel of 36 human cancer cells, with **45** exerting the highest cytotoxic effect in the low nanomolar range (i.e., IC_50_ value of 26 nM against breast cancer MAXF 401NL). As **47** and **49** lacked a significant cytotoxic action, it could be argued that modifications at C-1 are crucial for bioactivity. Hydroxymethyl and methyl substituents allowed to keep cytotoxicity while the carboxylic group abolished any effect. Furthermore, the potential antitumor activity of **45** and **46** was related to proteasome inhibition, as suggested by in vitro enzymatic-assays and the COMPARE analysis of the cytotoxic selectivity pattern of **46**. While the cytotoxic selectivity profile of **45** did not show any correlation with standard antitumor compounds, **46** exhibited the same selectivity pattern as the proteasome inhibitor tyropeptin A [66]. Interestingly, **45** exerted a similar extent of inhibition against all the three proteolytic sites of the 20S proteasome, whereas **46** was able to inhibit preferentially the chymotrypsin-like activity in enzymatic assays [67]. 

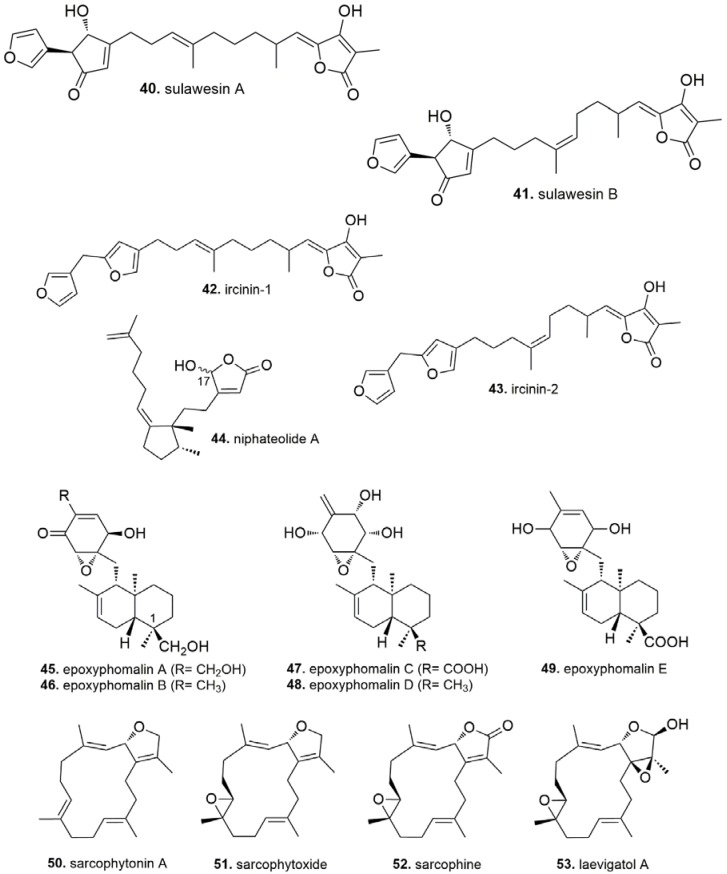



Using an EGFP-UL76 high-content screening assay, Ling and colleagues screened marine natural products from Formosan soft corals of the genus *Sarcophyton*, leading to the identification of four UPP inhibitors—namely sarcophytonin A (**50**), sarcophytoxide (**51**), sarcophine (**52**), and laevigatol A (**53**) [68]. Chemically, these compounds are monocyclic diterpenes displaying a cembrane-based scaffold. Compounds **50**–**53** exhibited a similar behavior as bortezomib and MG132, eliciting increase of EGFP-UL76 aggresome formation, due to impairment of proteasome degradation activity. These cellular effects were flanked by accumulation of polyubiquitylated proteins for compounds **50**–**52.** Notably, compounds **50** and **52** were shown to deregulate ubiquitylation of the proteasomal 19S S5a subunit in a different way from bortezomib, suggesting a distinct mechanism of action involved in UPP inhibition for the soft-coral derived compounds [68].

### 4.2. Alkaloids

Aaptamines are a group of benzo[*de*][1,6]-naphthyridine alkaloids commonly found in marine sponges of the genus *Aaptos*, displaying anticancer properties among other bioactivities. Tsukamoto et al. demonstrated the ability of aaptamine (**54**), isoaaptamine (**55**), and demethylaaptamine (**56**) to inhibit the chymotrypsin-like and caspase-like activities of the proteasome (with IC_50_ values ranging from 1.6 μg/mL to 4.6 μg/mL), while showing less inhibition of the trypsin-like activity of the proteasome [69]. **54**–**56** were able to exert a cytostastic/cytotoxic effect against HeLa cells, even if this action did not correlate with their efficacy in proteasome inhibition, thus suggesting concurrent mechanisms were involved in their mode of action. It has been observed **54** induces a p21-dependent G2/M cell-cycle arrest, where increased p21 expression could be related to proteasome inhibition. In a subsequent study, aaptamines were shown to exert similar anticancer effects in cisplatin-sensitive and -resistant human embryonal carcinoma cell lines NT-2 [70]. Despite **54**, **55**, and **56** promoted different alterations in protein expression, gene ontology analyses identified myc, p53, and TNF as putative targets or downstream effectors of aaptamines in NT-2 resistant cells. Further investigations about the mechanism of action of **54**–**56** enabled detection of (a) enhanced levels of AP-1 and NF-κB transcriptional activities and (b) unaffected (for **54**) or downregulated (for **55** and **56**) p53 transcriptional activity upon treatment with aaptamines at high nontoxic concentrations [71]. Interestingly, at low nontoxic concentrations, a significant inhibition of EGF-induced JB6 cell transformation could be appreciated, unveiling cancer preventive properties for **54**–**56** [71]. Recently, in vitro and in vivo assays shed light on a dual-induction of p21 and p27 by aaptamine alkaloids, responsible for G0/G1 cell cycle arrest and apoptosis specifically in t(4;11) leukemia cells [72].

The tetrahydroisoquinoline-alkaloid salsolinol (**57**) was isolated from the Indonesian sponge *Xestospongia* cf. *vansoesti* together with three analogues—namely norsalsolinol (**58**), *cis*-4-hydroxysalsolinol (**59**), and *trans*-4-hydroxysalsolinol (**60**)—in the frame of a research program aiming to discover novel lead compounds as proteasome inhibitors [73]. Compounds **57** and **58** impaired the chymotrypsin-like activity of the 20S proteasome preparation from human erythrocytes, with IC_50_ values of 50 μg/mL and 32 μg/mL, respectively, while **59** and **60** were inactive. For **57** and **58**, this biological effect was flanked by cytotoxicity against human cervix epithelioid carcinoma HeLa cells at high micromolar levels. Furthermore, **57** exerted cytotoxic action against murine leukemia (L1210), human amnion (FL), human oral epidermoid carcinoma (KB), and human lung adenocarcinoma (A549) cell lines. Interestingly, **57** was previously detected in mammalian brains where it has been recognized to act as a neuromodulator [74]; nonetheless, this compound is neurotoxic and is involved in the etiopathogenesis of Parkinson’s disease at non-physiological concentrations, probably due to its ability to induce dopaminergic neuron death through inhibition of the proteasome.

Hyrtioreticulins A (**61**) and B (**62**) were obtained after purification of the organic extract from the Indonesian sponge *Hyrtios reticulatus*. Compounds **61** and **62** are epimeric tetraydro-β-carboline alkaloids exhibiting ubiquitin-activating enzyme (E1) inhibition with IC_50_ values of 2.4 μM and 35 μM, respectively [75]. After binding ATP and ubiquitin in the relevant binding sites, E1 enzymes catalyze adenylation of ubiquitin, which is subsequently transferred to a cysteine-residue in the active site of E1 through formation of a thioester bond. Mechanistically, hyrtioreticulins have been shown to interfere with E1 activity after ubiquitin has been accommodated in its binding site, differently from himeic acid, which hampers ubiquitin recognition by E1 [76]. The *trans* configuration at C-1 improves E1 inhibition of **61** compared to **62**, while the absence of the imidazole ring (as observed in hyrtioreticulin E, which displays a methyl group instead of the imidazole) results in the loss of bioactivity [75]. Although being a highly potent E1-inhibitor in enzymatic assay, it is worth mentioning that **61** displayed weak cytotoxicity against HeLa cells.

Evaluation of proteasome inhibitory activity of three spongean oroidin-derived alkaloids was the object of the work of Lansdell and colleagues. These molecules—namely palau’amine (**63**), dibromophakellin (**64**), and dibromophakellstatin (**65**)—were found to inhibit the chymotrypsin- and caspase-like activities of the 26S proteasome as well as the chymotrypsin-like activity of the immunoproteasome, in the low micromolar range (2.3–27.1 μM), while lacking any relevant effect against the trypsin-like proteolytic site [77]. This behavior may at least partly explain the cytotoxic properties of these compounds. The natural (−)-enantiomer of **63** was the most bioactive compound and binding of **63** to the 20S proteasome was consistent with an irreversible inhibitory mechanism, according to kinetics data and washing experiments. Deeper investigations revealed that **63** induced ubiquitinylated proteins accumulation and prevented IkBα degradation in HeLa cells, through negative modulation of the proteasome [77].

Spongiacidin C (**66**), hymenialdisine (**67**), and debromohymenialdisine (**68**) are pyrrole alkaloids derived from the Indonesian sponge *Stylissa massa* [78]. Although being closely related from a structural point of view, only **66** exhibited a significant and rather selective inhibition of the deubiquitylating enzyme USP7 (IC_50_ = 3.8 μM) in a panel of five different USP family members (USP7, USP2, USP8, USP21, and SENP1). The hydantoin ring in **66** is essential for bioactivity, as replacing it with an aminoimidazolinone ring weakened dramatically inhibitory properties of **67** and **68**. In follow-up cytotoxic assays, compound **66** was unable to affect proliferation of the p53 wild type, USP7 expressing human colorectal cancer cell line HCT-116 even at 50 μM concentration [78].

Manzamines are a large family of complex polycyclic alkaloids, possessing a fused and bridged tetra- or pentacyclic ring system, which is usually attached to a β-carboline moiety. Recently, purification of the chemical extract of *Acanthostrongylophora ingens* (Indonesia) afforded two further members of the manzamine family, acantholactam (**69**) and pre-*neo*-kauluamine (**70**), along with manzamine A (**71**) and *neo*-kauluamine (**72**) [79]. Acantholactam displays an hexenoic-acid moiety linked to the γ-lactam ring, probably derived from oxidative cleavage of the hexahydroazocine ring of **71**; *neo*-kauluamine is likely a dimerization-product of **70**. Among these compounds, **72** was found to act as the most potent inhibitor of the chymotrypsin-like activity of the 20S proteasome particle (IC_50_ = 0.13 μM) and as the most effective cytotoxic agent against HeLa cells (IC_50_ = 5.4 μM). A certain degree of these biological effects was also detected with compounds **70** and **71**, but bioactivity was dramatically lowered in assays based upon treatment with **69**, thus revealing that the hexahydroazocine ring is an essential part of the pharmacophore of these molecules.

Further investigations of the metabolome of *Acanthostrongylophora ingens* expanded the manzamine family, yielding four new proteasome inhibitors (**73**–**76**) [80]. Acanthomanzamines A (**73**) and B (**74**) were identified as the first manzamine-related alkaloids where the β-carboline moiety is replaced by a tetrahydroisoquinoline ring, while acanthomanzamines D (**75**) and E (**76**) possess an additional oxazolidine and 2-methyloxazolidine ring, respectively, fused to the canonical manzamine backbone. SAR observations revealed two clear bioactivity trends: (1) tetrahydroisoquinoline-acanthomanzamines were more cytotoxic than the β-carboline derivatives against HeLa cells; (2) on the other hand, the latter compounds were more effective as inhibitors of the chymotrypsin-like proteolytic site. Overall, enzymatic assays with manzamine-alkaloids **69**–**76** highlighted that combination of an intact hexahydroazocine ring and a β-carboline moiety enhanced proteasome inhibitory activity of manzamines.

Two β-carboline alkaloids, variabines A and B (**77**–**78**), were isolated from the Indonesian sponge *Luffariella variabilis* and evaluated in enzymatic assays for their ability to target the UPP pathways as modulators of E1, E2, E3, and chymotrypsin-like activities. Interestingly, it was reported that **78** repressed both the chymotrypsin-like activity of the proteasome and the Ubc13–Uev1A interaction, with IC_50_ values of 16 and 20 μM, respectively [81]. E1 and E3 activities were unaffected after treatment with **77** and **78**. In addition, sulfonation of the hydroxy-group at C6 in **77** lowered significantly E2- and proteasome-inhibitory activities.

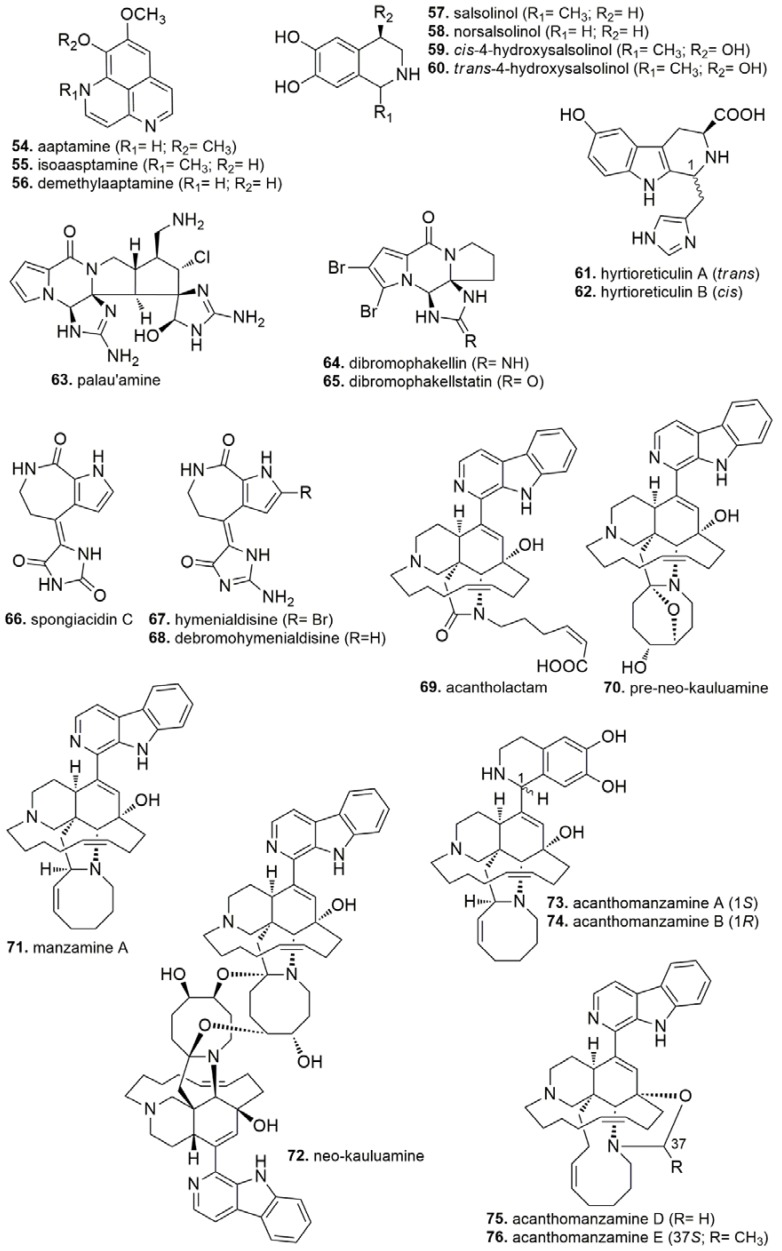

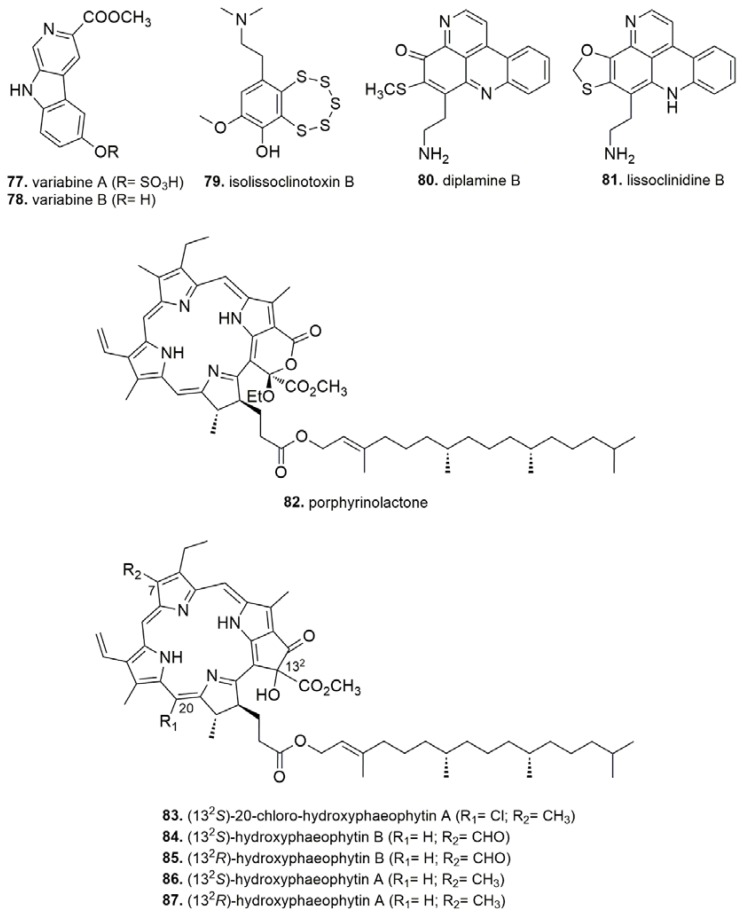





Clement et al. developed a high-throughput electrochemoluminescent screening method to detect the ubiquitin-ligase activity of the E3 enzyme HDM2 in natural product extracts. Using this method, bioassay-guided fractionation of the organic extract of the ascidian *Lissoclinum* cf. *badium* (Papua New Guinea) afforded a benzopentathiepin alkaloid, isolissoclinotoxin B (**79**), and two pyridoacridine alkaloids—diplamine B (**80**) and lissoclinidine B (**81**)—displaying HDM2-inhibitory activities with IC_50_ values of 58.6, 101.3, and 98.1 μM, respectively [82]. Although being toxic to retinal pigment epithelial (RPE) cells, compound **79** did not show the expected increase of p53 levels, which usually occurs after HDM2-inhibition. On the other hand, compound **80** underwent degradation easily over time in cell-based assays. Therefore, lissoclinidine B was selected for further investigation. Notably, **81** increased p53 and HDM2 levels more efficiently in cell-based assays than in enzymatic assays, unveiling that other mechanisms than the E3-inhibitory activity were involved in its action. Indeed, the highest E3-inhibitory activity was already observed at 10 μM of **81** [82]. Within the HDM2 pathway, it was demonstrated that **81** increased cellular levels of HDM2 and p53 through inhibition of HDM2-mediated ubiquitylation of HDM2 and p53, thus preventing their proteasome-degradation. More interestingly, this compound selectively killed transformed cell lines harboring wild-type p53, triggering apoptosis in a p53-dependent manner. Using mouse embryo fibroblasts (MEFs) and HCT116 as cell models, a marked decrease of wild-type p53 cell viability was detected at 20 μM while p53-deficient cells were resistant to drug-treatment [82].

An extensive analysis of the ethanolic extract from the green alga *Cladophora fascicularis* (China) allowed to identify six phaeophytins (**82**–**87**), that can be classified as alkaloids possessing a porphine skeleton and a long chain diterpene alcohol, namely phytol [83]. These compounds were determined as porphyrinolactone (**82**), 20-chlorinated (13^2^-*S*)-hydroxyphaeophytin A (**83**), (13^2^-*S*)-hydroxyphaeophytin B (**84**) and A (**86**), and (13^2^-*R*)-hydroxyphaeophytin B (**85**) and A (**87**). All the previous phaeophytins were shown to inhibit TNFα-activated nuclear translocation of NF-κB in HeLa cells at 50 μM, with **85** and **87** being the most potent among the tested compounds [84]. In addition, compounds were tested in vitro to provide a first evidence for the potential relationship between the inhibitory effect of NF-κB activation and proteasome inhibition. In enzymatic assays, phaeophytins revealed to inhibit the ChT-L activity of the 20S proteasome. Particularly, preliminary SAR emerged from these assays: (a) the phaeophytin-B-type molecules were stronger proteasome inhibitors than the phaeophytin-A-type compounds, suggesting a key role of the aldehyde group at C7 in drug–target interaction; (b) 13^2^-*R* epimeric phaeophytins exerted a stronger effect than 13^2^-*S* epimeric phaeophytins; (c) chlorination at C20 improved inhibitory activity of **83** compared to **86** [84]. It is worthy to mention that a positive correlation between NF-κB deactivation and proteasome inhibition was observed only in the case of **85**.

### 4.3. Peptides

The cyclic peptide scytonemide A (**88**) was isolated through bioassay-guided fractionation of the extract from the cultured freshwater cyanobacterium *Scytonema hofmannii* [85]. The core structure of this peptide contains an unusual imine bond, which could represent an electrophilic site for covalent binding with the side-chain hydroxy group of the N-terminal threonine residue of the proteasome. Although being a potent 20S proteasome inhibitor (IC_50_ = 96 nM vs. the ChT-L proteolytic activity) in enzymatic assays, **88** lacked antiproliferative effects against human colon cancer HT-29 cell line. It was demonstrated that **88** penetrated cell membrane and inhibited 20S proteasome activity in a luminescence-based cellular assay, displaying an 80% inhibition rate at 6.7 μM, a concentration where no cytotoxicity was observed. Chemical instability or early metabolic degradation of **88** could explain the failure of this compound in exhibiting a cytotoxic effect [85].

The cyclic depsipeptide largazole (**89**) was isolated from tropical marine cyanobacteria of the *Symploca* genus. Compound **89** contains a methylthiazoline-thiazole moiety in the peptidic core and a rare 3-hydroxy-7-mercaptohept-4-enoic acid unit, which is linked to an octanoate residue through a thioester bond. Largazole possesses highly differential antiproliferative activity at nanomolar levels, preferentially targeting transformed over non-transformed cells [86]. Beyond acting as an inhibitor of class I histone deacetylases [86], **89** was reported to inhibit the ubiquitin-activating enzyme E1 (IC_50_ = 29 μM) [87]. Particularly, **89** blocked the adenylation step of E1 activation in a nucleotide exchange assay. Furthermore, it was shown to reduce ubiquitylation of p21 and TRF1 in vitro, leading to their stabilization within cells. In addition, E1 inhibition was demonstrated to be not related to the histone deacetylase-inhibitory effect of **89**. Largazole appeared to be specific for the human ubiquitin pathway as it was relatively inactive towards the ubiquitin-like protein SUMO1 and the enzyme Uba1p, a homolog of E1 in *Schizosaccharomyces pombe*. Development of largazole analogues (Figure S3) unveiled that the intact macrocyclic peptidic core as well the presence of the hydrophobic side chain are crucial for the inhibition of the ubiquitin-activating enzyme E1, while the thioester bond is not essential [87].

Two novel α,β-epoxyketone proteasome inhibitors, namely carmaphycin A (**90**) and B (**91**), were found in trace amounts through NMR- and bioassay-guided purification of the extract from the marine cyanobacterium *Symploca* sp. [88]. Carmaphycin A displays a leucine-derived epoxyketone warhead linked to a methionine sulfoxide residue, in turn connected to a N-hexanoyl-valine unit. On the other hand, the sulfoxide group of **90** was replaced by a sulfone in **91**. Both compounds acted as irreversible proteasome inhibitors of the 20S proteasome from *Saccharomyces cerevisiae*, at nanomolar levels (IC_50_ = 2.5 nM). Notably, carmaphycins exerted potent antiproliferative effects against lung, colon, and brain cell lines in the low nanomolar range (1–50nM), with the most sensitive cell lines exhibiting mutations of KRAS and tp53 genes. Differently from other proteasome inhibitors, **90** and **91** were significantly more effective against solid tumor rather than leukemia cell lines. Through combination of molecular modelling and crystallographic studies, a comprehensive understanding of the proteasome-binding mode of carmaphycins was achieved [89]. The lipopeptidic core is involved in key interactions to form a stable anti-parallel beta sheet within the ChT-L binding site and positions correctly the epoxyketone warhead for covalent binding of the Thr1 proteasome catalytic residue. The inhibition process consists of a two-step mechanism (Figure 3a). Initially a 1,2-addition of the γ-OH group of Thr1 to the carbonyl group of the epoxyketone leads to the formation of a reversible hemiketal derivative; then, the α-amino-group of Thr1 makes a nucleophilic attack at C-2 of the epoxide moiety to give a stable morpholine adduct [89].

Fellutamides A–F (**92**–**97**) are lipopeptide aldehyde inhibitors of the human proteasome and come from different fungi, including some marine species (*Penicillium fellutanum*, *Aspergillus versicolor*). Chemical structures of fellutamides (classified according to Pirrung et al.) mainly differ for the length of the N-terminal fatty-acyl chain and the side chain group of the C-terminal amino acid residue [90]. Fellutamides were described as highly cytotoxic compounds in the literature. Fellutamide B (**93**) was reported to have antiproliferative activity against sarcoma, glioblastoma, fibroblast, glioma, leukemia, epidermoid carcinoma, and pheochromocytoma cell lines through induction of cell-cycle arrest [91]. In addition, fellutamides D (**95**) and E (**96**) were reported to be inactive against A549 lung carcinoma cells, but to inhibit PC3 prostate cancer cell-growth by inducing G2/M phase arrest and apoptosis [92]. Human skin cancer cells (SK-MEL-2), CNS cancer cells (XF498), and colon cancer cells (HCT15) were very sensitive to treatment with fellutamide F (**97**), which was as potent as doxorubicin against these cell lines [93]. In enzymatic assays, **93** inhibited the chymotrypsin-, caspase-, and trypsin-like activities of the human proteasome with IC_50_ values of 9.4 nM, 1.2 μM, 2 μM, respectively [91]. A significant downregulation of the ChT-L proteolytic capacity was also observed for **95** and **96** [92]. The crystal structure of the yeast 20S proteasome in complex with fellutamide B provided insights into the inhibitory mechanism of **93** [91,94]. The aldehyde group of **93** undergoes nucleophilic attack by the γ-OH group of Thr1 of the proteasome, thus yielding a hemiacetal adduct, which in turn forms a stabilizing hydrogen bond with the free amino group of Thr1 (Figure 3d). Further interactions of the peptidic core of **93** are engaged to stabilize the inhibitor-proteasome complex [91]. Notably, the long hydrophobic tail of **93** was found to assume different orientations according to the proteolytic site, while the peptidic skeleton adopts very similar conformations in all the three catalytic sites. Interestingly, only in the ChT-L proteolytic site, the C24—C29 fragment of the fatty acyl chain is accommodated in a hydrophobic groove of the proteasome, which is formed during ligand binding, thus highlighting a certain degree of plasticity of the proteasome [91]. Kinetic studies unveiled that **93** followed a reversible two-step slow binding mechanism for proteasome inhibition [94].

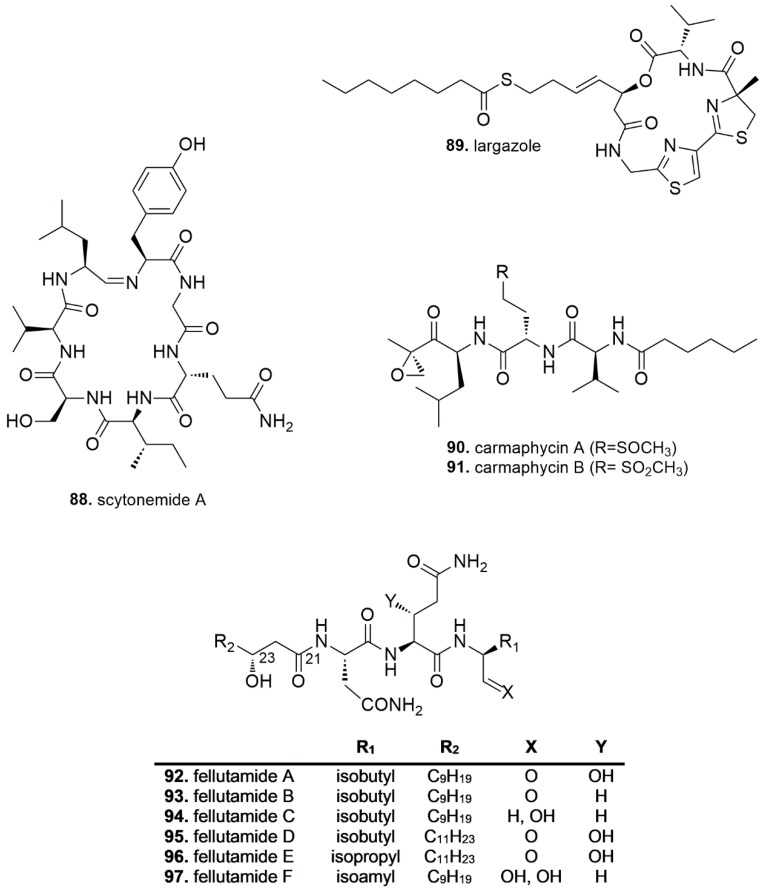



### 4.4. Sphingolipids and Phospholipids

Leucettamol A (**98**) is an α-ω bifunctionalized sphingolipid found in the Indonesian sponge *Leucetta microrhaphis*. This compound was identified as the first inhibitor of the formation of the heterodimer complex between the ubiquitin-conjugating enzyme Ubc13 and Uev1A in ELISA assay, with an IC_50_ value of 106 μM [95]. Interestingly, leucettamol was unable to inhibit formation of the Ubc13/MMS2 complex, although Uev1A and MMS2 share approximately 90% amino acid sequence identity. These results suggested a selective E2-inhibitory action of **98**. In addition, preparation and bioevaluation of a fully reduced (**99**) and a tetraacetate (**100**) derivatives of leucettamol allowed to achieve some clues about SAR [95]. Hydrogenation of **98** improved bioactivity as **99** had a 12-fold higher potency; on the other hand, fully acetylation of **98** yielded a completely inactive compound (**100**), unveiling that free hydroxy and/or amino groups are crucial for drug–target interactions.

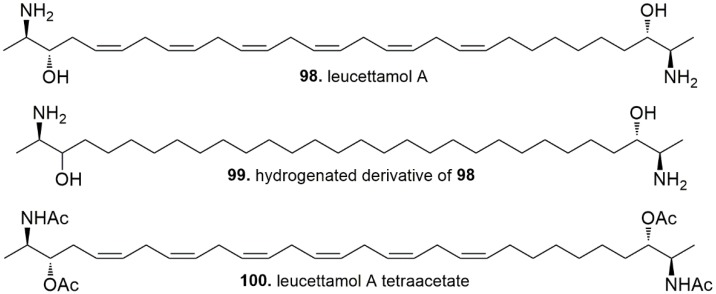



Siladenoserinols A–L (**101**–**112**) were recovered after chemical purification of the butanolic extract from a tunicate of the family *Didemnidae* (Indonesia). Their chemical structure features a sulfonated serinol moiety and a characteristic 6,8-dioxabicyclo [3.2.1]octane unit, together with either a glycerophosphocholine or a glycerophosphoethanolamine residue [96]. All compounds **101**–**112** resulted to be E3 inhibitors, interfering with HDM2-p53 interaction at low micromolar levels in ELISA assays (IC_50_ values ranged from 2.0 μM to 55 μM). Siladeserinol A (**101**) and B (**102**) were the most potent inhibitors among these compounds. Overall, the observed bioactivity trends clearly indicated that (a) the sulfamate derivatives were more active than the corresponding sulfate derivatives, and (b) the acetyl derivatives exhibited stronger inhibition than the corresponding hydroxy derivatives. In in vitro cytotoxicity assays, viability of A549 cancer cells was significantly reduced (80%) after treatment with 10 μM of **101** [96].

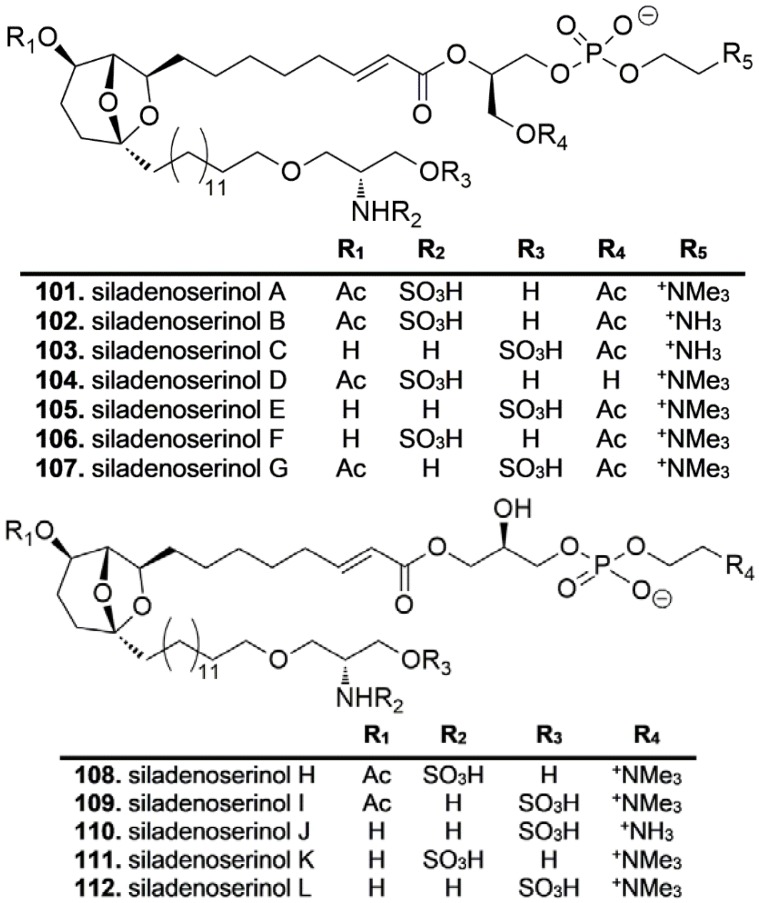



### 4.5. Sterols

Following discovery of **98**, two further compounds (**113**–**114**) inhibiting formation of the Ubc13/Uev1A were isolated from the sponge *Lissodendryx fibrosa* (Indonesia)*.* Manadosterol A (**113**) and B (**114**) are two sulfonated dimeric sterols, exhibiting higher activity than leucettamol, with IC_50_ values of 0.09 μM and 0.13 μM, respectively [97].

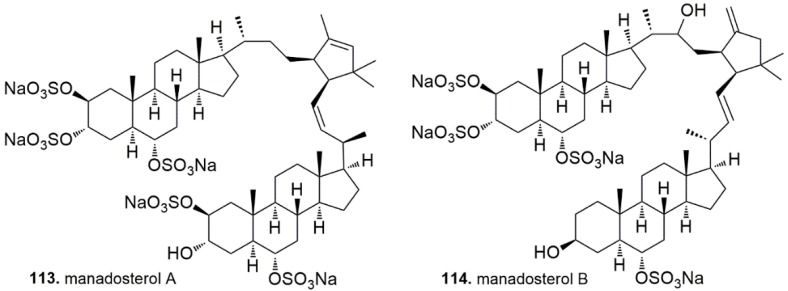



### 4.6. Quinones

Fifteen cyclic quinones (**115**–**129**), isolated from the Indonesian sponge *Petrosia alfiani*, were identified as USP7 inhibitors in enzymatic, fluorescence-based bioassays [98].

Petroquinones A (**115**) and B (**116**) are composed of three pentacyclic xestoquinone (**128**) units; petroquinones C-H (**117**–**122**) are dimeric xestoquinone-derivatives; petroquinones I-L (**123**–**126**) feature a xestoquinone scaffold fused with thiomorpholine 1,1-dioxide and pyrrolidine-2,4-diol moieties; **127** and **129** were shown to be 1-(2-hydroxyethyl)xestoquinone and halenaquinone, respectively. Except for **118** and **123**–**126** (IC_50_ > 5μM), all compounds exerted a strong inhibitory action against deubiquitinase activity of USP7 enzyme, with IC_50_ values in the range of 0.13–2.0 μM [98]. Xestoquinone (**128**) demonstrated to be the most active among these quinones. The putative inhibitory mechanism of quinones **117**, **119**–**122,** and **127**–**129** could involve C14 or C15 of the quinone moiety, which is expected to react with the catalytic sulfhydryl group in the active site of USP7. With respect to **115** and **116**, this hypothetical mechanism cannot occur as C14 and C15 are embedded in the polycyclic structure of the molecules and, therefore, **115** and **116** must interact with the target in a different way [98].

A sponge specimen of the genus *Xestospongia* sp. (Indonesia) provided a number of quinones, including halenaquinone (**129**), a hydroxyethyl derivative of halenaquinone (**130**), and 3-ketoadociaquinones A (**131**) and B (**132**) [99]. These compounds were evaluated for their inhibitory activity against the chymotrypsin-like catalytic site of the proteasome, with **129** and **130** being strong inhibitors at very low micromolar concentrations (IC_50_ values of 0.63 μM and 0.19 μM, respectively). Conversely, **131** and **132**, featuring a thiomorpholine 1,1-dioxide, were weakly active even at 5 μM.

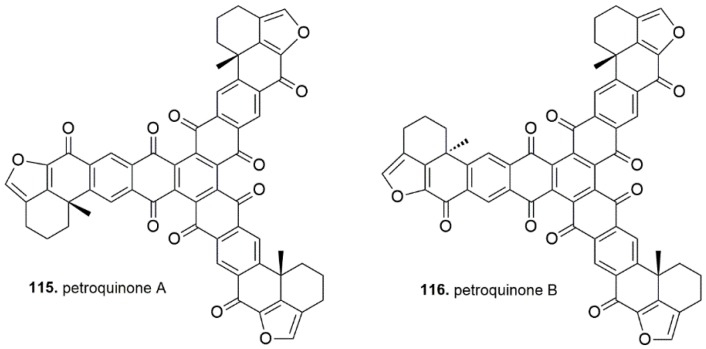

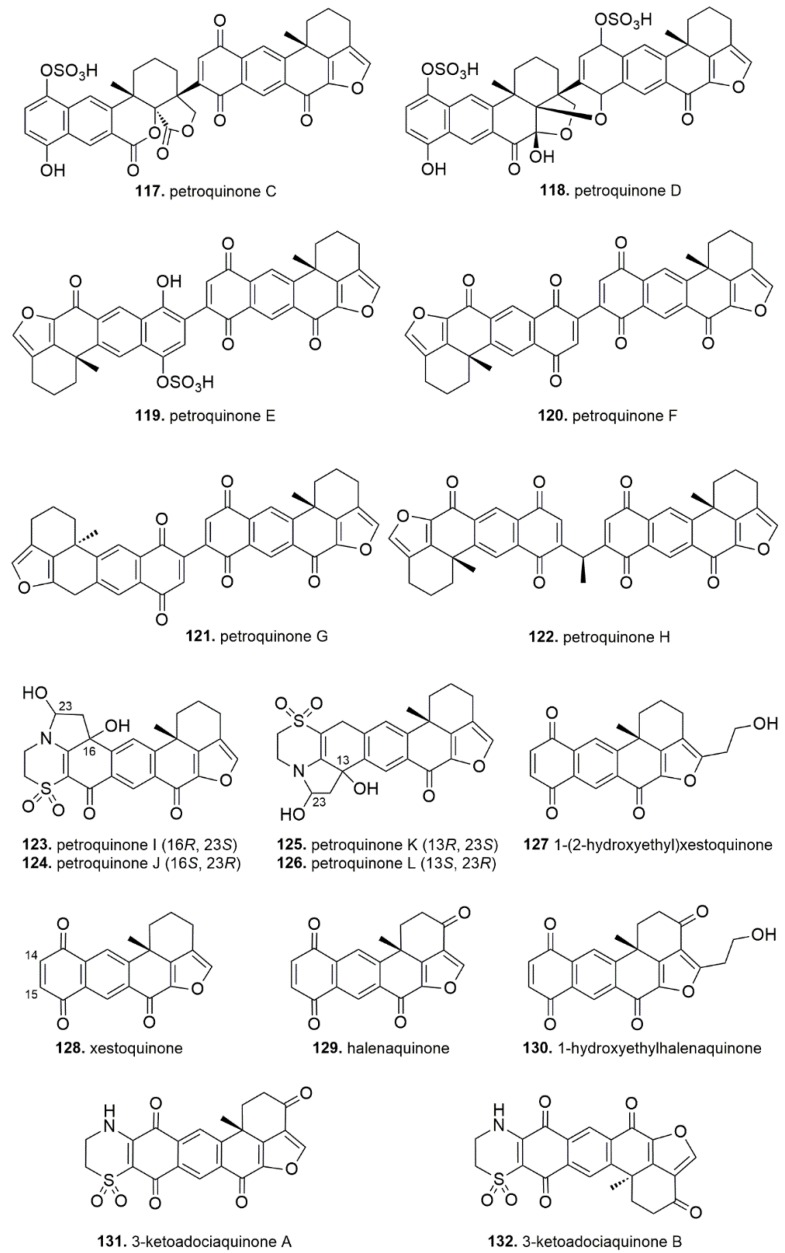





## 5. Resistance Mechanisms to Proteasome Inhibition

The beneficial effects of proteasome inhibition in patients with multiple myeloma and mantle cell lymphoma were unfortunately not observed along treatment of other hematological malignancies (such as acute myeloid leukaemia, myelodysplastic syndrome, and acute lymphoblastic leukaemia) and solid tumors [100,101]. Solid tumors exhibit a primary resistance to proteasome inhibitors, while acquired resistance can be developed in patients with myeloma and mantle cell lymphoma even after a good initial response. Overall, a deeper understanding of resistance mechanisms is required to pave the way for combination therapy based upon proteasome inhibitors and drugs able to sensitize resistant cancer cell phenotypes and/or block adaptive responses induced by proteasome inhibition.

Plasma cells are undoubtfully the most sensitive cell type to downregulation of the UPP, as they have to sustain high rates of immunoglobulin synthesis. This is even more true for blood cancer cells, which are characterized by an aberrant production of proteins. The increased protein synthesis dramatically affects the fine balance between proteasome load and capacity, making malignant plasma cells more sensitive to proteasome inhibition than normal cells [102]. This selective action can be also explained considering that the NF-κB pathway is constitutively activated in myeloma cells and the enhanced IkB-mediated inhibition of NF-κB is one of the main downstream effects of proteasome inhibition [103]. In addition, myeloma cells are more sensitive to proteasome inhibition as they accumulate higher amounts of misfolded proteins, leading to a proteotoxic state which promotes apoptosis and UPR response [104].

To date, several preclinical studies were addressed to elucidate acquired and inherent resistance mechanisms to proteasome inhibition, most of these studies being based upon treatment with bortezomib. Overall, cell responses hampering proteasome inhibition are directed either to improve proteasome capacity or reduce proteasome load, thereby promoting cancer cell survival.

In vitro assays revealed resistant cell lines developed mutations in the binding pocket of the subunit β5 of the 20S proteasome, which could negatively affect the reversible binding of bortezomib [105]. However, such mutations were not detected in bortezomib-resistant patients [10]. Induction of expression of proteasome subunits β5, β2, and β1 was observed in myeloma cell lines trying to compensate proteasome inhibition, even if this adaptive response was too weak to fully explain the acquired resistance by treated cells [106]. The antioxidant response pathway was reported to be activated in resistant cells, leading to induction of the proteasome maturation protein (POMP), which is essential to assemble proteasome subunits, and, therefore, improve its degradation capacity [107]. Furthermore, the proteasome-inhibitor-resistant phenotype was shown to enhance also the EGFR signaling cascade to increase production of proteasome subunits [108].

Impairing proteasome activity induced also expression of heat-shock proteins and chaperones to improve protein folding, reduce proteotoxic stress, and restore proteasome load/capacity ratio, thereby raising the apoptosis threshold [109]. Similarly, proteasome inhibition forced cells to activate alternative proteolytic processes, such as the aggresome-autophagy pathway, to reduce the ‘proteasomal stress’, sequester deleterious proteins in aggresomes, and guarantee cell survival [110]. Notably, Leung-Hagesteijn et al. discovered that myeloma cells with low expression levels of IRE1 and XBP-1 displayed less differentiation, decreased immunoglobulin synthesis, lower proteasomal stress and innate resistance to bortezomib, compared to other tumor subpopulations which were sensitive to bortezomib [111]. In bortezomib-resistant myeloma cell models, induction of IGF-dependent AKT signaling promoted an antiapoptotic state within cells. Cotreatment with bortezomib and an IGF1R inhibitor resulted in re-sensitization of these cancer cells to bortezomib and increased cell death [112].

Clinical studies revealed bortezomib to be ineffective in treatment of solid tumors. A first hypothesis to explain this resistance was based upon (1) insufficient tumor penetration, related to tumor architecture and/or limited perfusion; and (2) pharmacokinetic and pharmacodynamic properties of this drug, which appears to be distributed mainly to blood and bone marrow rather than other tissues [10,100,113]. Moreover, key proteasome target proteins in solid tumors are likely to be different from those in hematological malignancies due to dependence on distinct oncogenic signaling pathways and the hypoxia-induced accumulation of oncoproteins [51]. As the three catalytic sites of the proteasome have different substrate selectivity, a broader and stronger proteasome inhibition rather than the reversible β5-specific inhibition of bortezomib could be necessary for treating solid tumors. In 2017, Weyburne and colleagues demonstrated that triple-negative breast cancer cell lines survived after exposure to doses of bortezomib or carfilzomib, which were able to block exclusively the β5 subunit of the proteasome [114]. Using the CRISPR gene editing, Weyburne et al. switched off the β2 and β1 proteolytic functions of the proteasome to show that ablation of the β2 activity was enough to re-sensitize breast cancer cells to bortezomib and carfilzomib. These data were confirmed by chemical inhibition of the β2 catalytic site [114]. It can be deduced that residual proteolytic β2-activity contributes significantly to cell survival in solid tumors after blockade of the β5 subunit, thus accounting for the observed intrinsic resistance.

The adaptive hyperactivation of β2 and β1 proteolytic functions was also observed after treatment of multiple myeloma and solid tumor patients with salinosporamide A, even if this drug can recognize all three β catalytic sites of the 20S proteasome. This compensatory response occurred after β5-inhibition upon initial dosing with salinosporamide A and was related to an allosteric interaction between the different proteasome subunits rather than overexpression of new proteasomes, as expected for an irreversible inhibitor [51]. However, differently from bortezomib, Levin et al. demonstrated that repeated dosing with salinosporamide A overcame the initial compensatory upregulation of β2 and β1 activities, thus resulting in a robust pan-proteasome inhibition due to the broader spectrum of the molecule [51]. Therefore, the irreversible mode of action of **1** as well as its ability to block all three β catalytic sites of the 20S proteasome suggest a novel approach to achieve efficacy against solid tumors too.

## 6. High-Throughput Screening for Discovery of Natural UPP Inhibitors

Differently from combinatorial chemistry products, natural products often feature complex structural architectures covering a more diverse chemical space and, therefore, represent a valuable treasure to discover novel biological activities. However, the whole purification process and sustainable production of bioactive natural compounds imply a time-consuming and often non-straightforward workflow. Different high-throughput screening (HTS) approaches have been developed to accelerate target-oriented fractionation of organic extracts leading to successful drug discovery. Herein, we summarize HTS methods specifically addressed to isolation of natural UPP inhibitors from heterogenous and complex extracts.

### 6.1. HTS Methods for Detection of Natural Proteasome Inhibitors

UV–vis spectrophotometric determination of proteasome activity is the most applied HTS for discovery of UPS-targeting molecules from crude extracts of natural sources. These assays are based upon non-natural peptides, featuring a C-terminal chromophore group, which generates a fluorescence signal after proteasomal-hydrolysis. The peptide sequence is designed to exhibit selective affinity for a specific proteasome subunit and, therefore, measure proteolytic activity of each catalytic site. The most commonly used chromophores are aromatic groups, including 2-naphtylamine, 7-amino-4-methyl-coumarin, and 4-methoxy-2-naphtylamine. This methodology is suitable for enzymatic- and cell- based assays and the fluorescence signal is directly proportional to the digestion capacity of the proteasome, allowing a fast, quantitative assessment of bioactivity [115]. In 2011, Götze and Saborowski described the application of NanoDrop fluorometry for measuring proteasomal enzymatic activities using fluorogenic substrates [116]. The main advantage of NanoDrop devices can be found in the reduction of amount of sample extracts, substrates, and cofactors to be used for an enzyme assay. However, UV–vis techniques are prone to a plenitude of artifacts caused by quenching and/or autofluorescence, which are quite common interferences when heterogeneous and colorful organic extracts are analyzed. In addition, a limiting factor with fluorescent substrates is that small adducts mimicking natural substrates and non-natural peptides may not be hydrolyzed efficiently.

An alternative HTS method relies on the use of a fluorescent-labeled proteasome inhibitor (fluorescent probe), which has to bind irreversibly all the three catalytic sites of the proteasome [115]. Aliquots of complex organic extracts are added to the assay mixture, including intact cells, cell-lysates, or purified proteasome. After incubation, the selected irreversible probe is added to the mixture and if the sample contains an inhibitory compound, the active sites of the proteasome are already blocked, thereby hampering labeling of the proteasome. Suppression of the fluorescence signal is indicative of proteasome inhibition and can be easily quantified. The fluorescence signals can be detected (a) through SDS-PAGE and fluorescence gel scanning devices for purified proteasomes and cell lysates, or (b) through confocal microscopy or flowcytometry if intact cells are used in the assay. This method excludes generation of false positives, as potential interfering compounds are removed through SDS-PAGE. However, this technique does not allow detection of reversible, non-covalent inhibitors of the proteasome as they are displaced by the competitive binding of the probe.

Recently, Stein et al. developed an NMR-based proteasome assay to detect inhibitory compounds in conglomerates [117]. This screening method uses a purified 20S core particle and a natural peptide substrate, which is selectively labeled through introduction of a carbonyl ^13^C probe at the scissile amidic bond. Proteolytic activity of the proteasome can be qualitatively and quantitatively evaluated by a peak shift in the ^13^C-NMR spectrum from 173 ppm to 177 ppm, corresponding to the intact amide educt and the hydrolyzed carboxylic acid derivative, respectively. NMR spectroscopy is suitable for analysis of crude mixtures as it is not affected by diffraction, autofluorescence or quenching as for UV–vis techniques. Moreover, the signal-to-noise ratio is considerably increased. In the carbonyl resonance area of the NMR spectra, putative overlapping NMR signals due to other compounds in the sample extract can be overlooked as natural compounds display only 1.1% of ^13^C carbon compared to the ^13^C-enriched probe used for the assay. The NMR-based approach allows screenings of almost 100 samples per day, as the recording time to acquire a single spectrum is about 15 min.

### 6.2. HTS Methods for Detection of Natural Inhibitors Targeting E1, E2, E3, and DUBs Enzymes

The ubiquitination cascade starts with the ATP-dependent loading of the E1-ubiquitin activating enzyme. E1 enzymes use ATP to adenylate the C-terminus of ubiquitin, yielding a ubiquitin-adenylate adduct and pyrophosphate. Then, an active site cysteine within E1 cleaves the ubiquitin-AMP ester bond, forming an E1-Ub intermediate through a thioester bond between the C-terminus of UB and the sulfhydryl of cysteine. Ub is transferred from the E1 to another active site cysteine within the E2-ubiquitin conjugating enzyme. The E3 ligase binds to both the E2 and the substrate, leading to isopeptide bond formation between Ub and a lysine residue of the protein substrate.

The most widely applied method to measure E1 activity is monitoring the formation of E1-Ub intermediate by semi-quantitative Western blotting analysis. Generally, epitopes (e.g., FLAG) or fluorescent-tags are attached to E1 enzymes or ubiquitin to have accurate quantification and increase sensitivity [75,87]. This technique can be easily adapted for evaluation of ubiquitin-E2 thioester bond formation, using an assay mixture including an ATP- and Ub-pre-charged E1 enzyme and a specific purified E2 protein [87]. However, western blotting analyses are time-consuming assays and high variability in signal detection may occur between different experiments.

With regard to evaluation of E2-ubiquitin conjugating activity, a 96-well ELISA assay has been set up to evaluate inhibition of the Ubc13-Uev1A (or MMS2) interaction, using human-Ubc13-coated multiwell plates and FLAG-Uev1A (or MMS2) [95]. Similarly, the ELISA method has been applied to monitor interaction between the transcription factor p53 and the E3 ligase HDM2, using the human p53 to coat the 96-well plates, the native HDM2 and a commercially available anti-HDM2 antibody for quantitative assessment of inhibitory activity [96].

In 2008, Sasiela and colleagues developed a high-throughput electrochemiluminescent assay to discover HDM2 inhibitory compounds from natural product extracts [118]. This system estimates E3-ligase activity of HDM2 measuring its autoubiquitylation capacity. Particularly, the HDM2 assay is based upon detection of polyubiquitylated Hdm2 by electrochemiluminescence generated by a ruthenium labeled polyubiquitin antibody. Assay samples, ubiquitin, and a pre-charged E1/E2 solution are added to the multiwell-plates containing Hdm2, and, after 30 min, the ruthenium-labeled polyubiquitin antibody is added too to record luminescence by a plate-reader. The electrochemiluminescent platform ensures an extremely sensitive detection and photons generated by Ruthenium (II) tris-2,2′-bipyridine-tag emit at a longer wavelength (620 nM) than most natural products.

In 2011, an innovative spectrophotometric assay has been set up by Berndsen and Wolberger for measuring ubiquitin transfer, without using tagged substrates or gel electrophoresis technique [119]. Even if this method has not been directly tested for screening of natural products mixtures, the authors assess this method to be amenable for HTS approaches. The assay correlates ubiquitin transfer by E2 and E3 to the E1-Ub intermediate formation; this is an ATP-dependent reaction releasing pyrophosphate, which subsequently undergoes hydrolysis to yield inorganic phosphate by a pyrophosphatase added in the assay solution. The inorganic phosphate is quantified by measuring the absorbance of molybdenum blue, a phosphomolybdate complex, that is formed from free phosphate by adding molybdate in the presence of ascorbic acid. For HTS applications, a plate reader capable of measuring molybdenum blue absorbance between 660 nm and 850 nm is needed.

HTS assays to evaluate proteolytic activity of DUBs enzymes have been developed along years, too. Many procedures currently in use rely on cleavage of linear Ub or small peptides mimicking the C-terminal part of Ub, fused to a fluorogenic substrate (e.g., AMC). In order to monitor deconjugation of Ub by USP7 during fractionation of sponge extracts, Yamaguchi and colleagues employed a recombinant USP7 protein and Ubiquitin-rhodamine 110 as quenched, fluorescent substrate [78]. USP7-cleavage of the amide bond between the C-terminal glycine of Ub and rhodamine results in an increase in rhodamine fluorescence at 535 nm (excitation wavelength 485 nm). In addition, Goldenberg et al. developed an HTS assay platform consisting of Ub fused to the reporter enzyme PLA_2_ (phospholipase A_2_) [120]. Isopeptidase activity of DUBs releases active PLA_2_ that cleaves its substrate, giving a readily quantifiable fluorescent response. The phospholipid *β*-BODIPY C5-HPC [2-(4,4-difluoro-5,7-dimethyl-4-bora-3a,4a-diaza-*s*-indacene-3-pentanoyl)-1-hexadecanoyl-*sn*-glycero-3-phosphocholine] and sphingolipid NBD C6-HPC {2-[6-(7-nitrobenz-2-oxa-1,3-diazol-4-yl)-amino]hexanoyl-1-hexadecanoyl-*sn*-glycero-3-phosphocholine} are fluorescent substrates commonly used for this assay, producing a fluorescence response over a wavelength range (500–650 nM) outside the UV range where most natural leads are able to emit.

### 6.3. Indirect Methods to Detect Natural Products Targeting the UPP Pathways

Differently from the above described approaches to isolate natural products blocking specific targets, several other non-specific methods have been developed to detect bioactive compounds through evaluation of downstream effects produced by UPP inhibition in cell-based systems. These methods allow to observe alterations of substrate levels which can be related either to a selective inhibition of an unknown target within the UPP pathway or to a broader variety of drug-induced mechanisms. When conglomerates are screened, trace-amount compounds risk to be overlooked due the detection limits of these approaches. Moreover, in some cases drug-treatment does not necessarily produce significant alterations in cellular responses even if the compound inhibits the UPP pathway at some point. Therefore, unspecific screening methods are usually recommended to support and complement target-oriented isolation of bioactive natural products from crude extracts. The main advantage of indirect methods can be found in the potential detection of compounds which are able to cross cell membrane and produce a certain cellular effect.

Inhibition of the UPP may result in accumulation of polyubiquitinated proteins. Ub accumulation assays allow to measure levels of Ub-labeled substrates within mammalian cell lines after drug-exposure [60,77]. After treatment, cell lysates are separated through SDS PAGE and ubiquitinated proteins are visualized by Western blotting analyses. Alternatively, UPP proteolytic activity can be monitored by measuring cellular degradation of Ub-tagged reporter proteins after treatment with a pure compound or an heterogenous sample. These assays rely on the use of transfected cell lines harboring a stable expression system encoding for the fusion construct Ub-green fluorescent protein (Ub-GFP) or Ub-luciferase [121,122]. Accumulation of the reporter protein due to decreased UPP digestion capacity can be measured by either spectrophotometry or fluorescence microscopy. In 2018, Ling and colleagues validated a novel, sensitive high-content screening assay to monitor aggregations of ubiquitinated proteins within cells expressing the human cytomegalovirus protein EGFP-UL76. This method is based upon high-content measurements of EGFP-UL76 aggresome formation by fluorescence imaging [68]. 

Screening techniques have been also addressed to quantify specific proteasomal substrates other than Ub. As explained above, impairing proteasomal activity usually leads to inhibition of IkB degradation and inactivation of the nuclear factor NF-κB as well as to stabilization of tumor suppressor factors, such as p53. Therefore, measurements of cellular IkB or p53 concentration levels by immunoblotting have been regarded as valuable approaches to discover UPP inhibitors [59,82].

## 7. Conclusions and Perspectives

The UPP pathway is the main regulator of protein turnover in eukaryotic cells, and its therapeutic modulation through proteasome inhibition has represented a definite step forward in treatment of hematological malignancies. Impairing proteasome activity in cancer cells has provided the chance to shift the fine balance between protein synthesis and degradation towards cell death, as demonstrated by recent introduction of bortezomib and carfilzomib in clinical settings.

**Figure 5:**
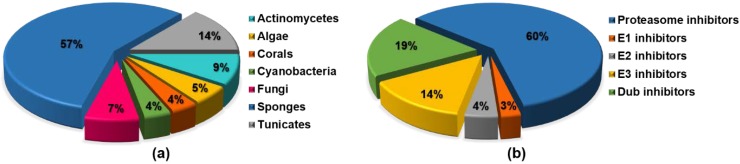
(**a**) Percentage distribution of natural UPP inhibitors discovered from marine sources in the last decade (2007–2018); (**b**) Distribution of pharmacological targets of reviewed UPP inhibitors.

Marine sources have been demonstrated to be an unexhaustive factory of UPP inhibitors, covering a wide chemical space. During the last decade, most of marine UPP inhibitors have been isolated from sponges or sponge-associated microorganisms (Figure 5a).

Even if the majority of resistance mechanisms to proteasome inhibitors remains yet unknown, several studies unveiled putative pathways hampering successful outcomes of proteasome inhibition-based therapy. In this contest, combination regimens have been tested in order to overcome resistance and sensitize cancer cells to proteasome inhibition. On the basis of the complementary effects on signal transduction and protein regulatory pathways, combining proteasome and histone deacetylase (HDAC) inhibition has provided a synergistic anti-tumor effect in tumor cell lines from patients with melanoma, pancreatic, and lung cancer after coadministration of salinosporamide A and vorinostat (a FDA approved HDAC inhibitor) [49]. Overall, another potential approach could be to design a ‘dual inhibitor’, which blocks at the same time the proteasome and the pathway responsible for resistance to proteasome inhibition. If this may appear too challenging from a chemical-synthesis perspective, natural products have been already shown to act as potential ‘dual inhibitors’ in some cases. The robust pan-proteasome inhibition by salinosporamide A overcame successfully the resistance mechanism triggered by the residual β2 proteolytic activity [51], which makes solid tumors refractory to treatment with bortezomib/carfilzomib. Moreover, largazole has been demonstrated to inhibit both the ubiquitin-proteasome pathway and the HDAC, thereby providing the basis for a synergistic anti-tumor effect.

Beyond the proteasome, the UPP has multiple other druggable targets, such as E1, E2, E3, and DUBs. The majority of the reviewed molecules have been shown to act mainly against the proteasome, highlighting that identification of drugs that specifically inhibit deubiquitinases and upstream regulatory components of the protein-turnover machinery has lagged behind (Figure 5b). However, a future perspective can be found in the discovery of new compounds targeting effectors of the ubiquitin cascade other than proteasome, as these molecules might show antitumor activity alone, and/or synergistic cytotoxic effects. Notably, it is worth mentioning that variabines feature a simple β-carboline moiety which allow them to repress both the chymotrypsin-like activity of the proteasome and the Ubc13–Uev1A interaction. Similarly, halenaquinone was shown to act as a deubiquitinase and a proteasome inhibitor at the same time.

Differently from proteasome or E1 inhibitors, selective inhibition of a certain isoform of E2, E3, or DUB enzymes has the potential to target a limited set of substrates and modulate the specific pathway regulated by that enzyme, thereby leading to fewer side effects and a more suitable targeted therapy [35]. As an example, blocking exclusively MDM2 among the 600 known E3 ligases leads to stabilization of the tumor suppressor factor p53, as MDM2 is the main regulator of p53 cellular degradation.

## Supplementary Materials

The following are available online at http://www.mdpi.com/1660-3397/16/12/467/s1. Figure S1. Salinosporamide A and synthetic analogues with altered C-2 and C-5 substituents. Figure S2. Petrosaspongiolide M and benzothiophenyl synthetic analogues. Figure S3. Largazole and synthetic analogues.
