# Supplementary material for: Clogging the Ubiquitin-Proteasome Machinery with Marine Natural Products: Last Decade Update
Source: Mar Drugs. 2018 Nov 26;16(12):467. doi: 10.3390/md16120467 (PMC6316072; doi:10.3390/md16120467)
Supplement: Supplementary file 1 [file marinedrugs-16-00467-s001.pdf]

Supplementary Information

Clogging the Ubiquitin-Proteasome Machinery with Marine Natural Products: Last Decade Update

Gerardo Della Sala [1], Francesca Agriesti [1], Carmela Mazzoccoli [1], Tiziana Tataranni [1], Valeria Costantino [2,*], and Claudia Piccoli [1,3,*]

[1] Laboratory of Pre-Clinical and Translational Research, IRCCS-CROB, Referral Cancer Center of Basilicata, 85028 Rionero in Vulture, Italy; gerardo.dellasala@crob.it (G.D.S.); francesca.agriesti@crob.it (F.A.); carmela.mazzoccoli@crob.it (C.M.), tiziana.tataranni@crob.it (T.T.)

[2] The NeaNat Group, Department of Pharmacy, University of Naples Federico II, via D. Montesano 49, 80131 Napoli, Italy

[3] Department of Clinical and Experimental Medicine, University of Foggia, via L. Pinto c/o OO.RR., 71100 Foggia, Italy

* Correspondence: valeria.costantino@unina.it (V.C.); claudia.piccoli@unifg.it (C.P.); Tel.: +39-081-678-504 (V.C.); +39-0881-588-060 (C.P.)

Table of contents:

Figure S1. Salinosporamide A and synthetic analogues with altered C-2 and C-5 substituents p.2

Figure S2. Petrosaspongiolide M and benzothiophenyl synthetic analogues p.2

Figure S3. Largazole and synthetic analogues p.3

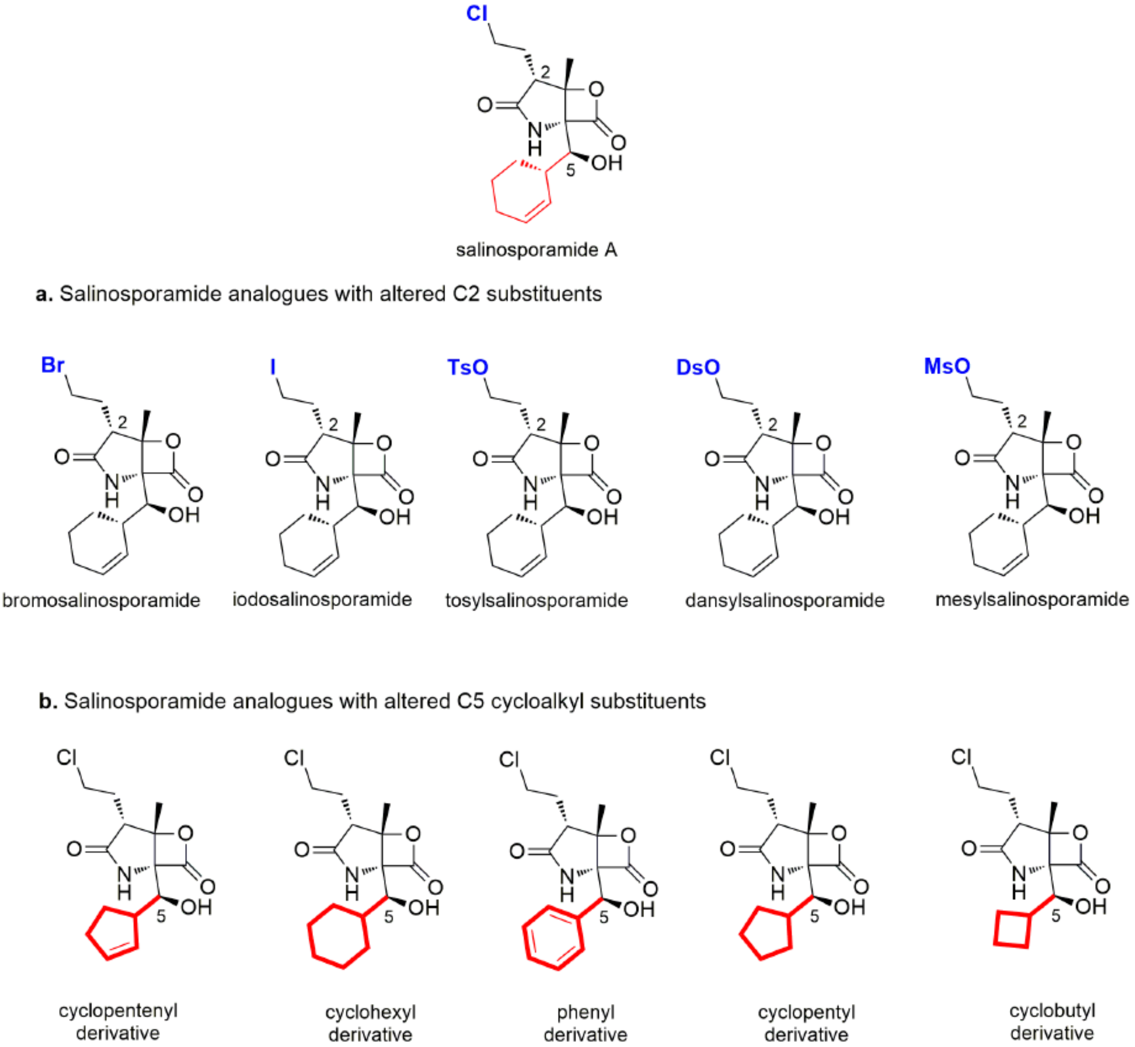

Figure S1. Salinosporamide A and synthetic analogues with altered C-2 and C-5 substituents.

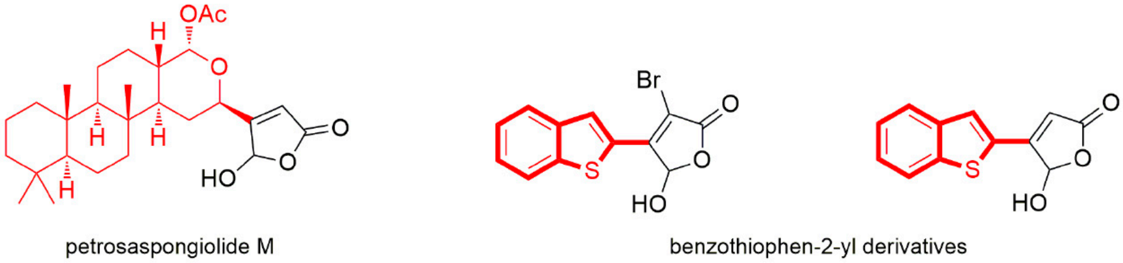

Figure S2. Petrosaspongiolide M and benzothiophenyl synthetic analogues.

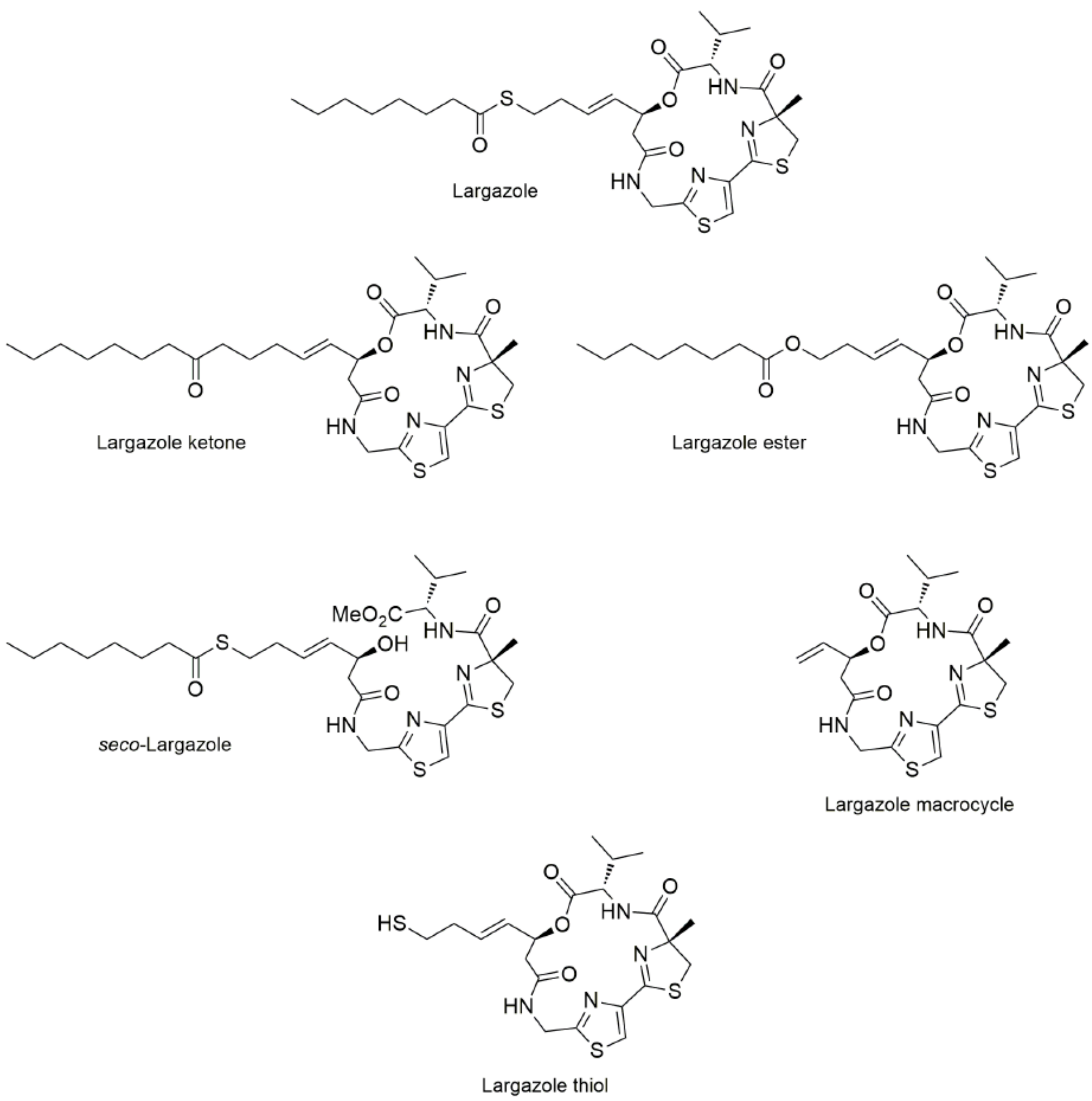

Figure S3. Largazole and synthetic analogues.
